# Global Potential Refugia and Conservation Gaps for Threatened Marine Rays Under Future Climate Change

**DOI:** 10.3390/ani16182924

**Published:** 2026-09-17

**Authors:** Tong Lu, Chengcheng Li, Weifeng Liu, Liang Liu, Tingting Li, Pipit Pitriana, Shenghao Liu

**Affiliations:** 1 Marine Ecology Research Center, First Institute of Oceanography, Ministry of Natural Resources, Qingdao 266061, China; lutong@fio.org.cn (T.L.); lichengcheng@fio.org.cn (C.L.); liuwf@fio.org.cn (W.L.); liuliang@fio.org.cn (L.L.); litingting@fio.org.cn (T.L.); 2 Research Center for Biosystematics and Evolution, Research Organization for Life Sciences and Environment, National Research and Innovation Agency, Cibinong 16911, Indonesia; pipitpitriana@yahoo.com

**Keywords:** Batoidea, cartilaginous fishes, biodiversity hotspots, occurrence records, species distribution modeling, spatial conservation prioritization

## Abstract

Climate change is redistributing marine biodiversity and may reduce the long-term effectiveness of static conservation networks. Threatened marine rays may be particularly vulnerable because many species have slow life histories, limited recovery capacity, and strong dependence on coastal and shelf habitats. However, the global distribution of habitats expected to remain suitable for threatened rays under severe future climate change, as well as their representation within existing marine protected areas, remain poorly understood. Here, we projected ensemble species distribution models for 85 threatened marine ray species for 2090–2100 under SSP5-8.5 and quantified habitat loss, persistence, gain, and net-richness change. Climate-resilient hotspots were identified from stable suitable-habitat richness and intersected with multi-source conservation-priority areas to delineate potential climate refugia. Habitat losses and negative net-richness changes were concentrated mainly in tropical and subtropical coastal waters, whereas gains were more extensive at higher latitudes. The Top 10% and Top 5% stable hotspots covered 6.47 and 3.89 million km^2^, respectively, yielding 1.64 million km^2^ of potential refugia and 1.06 million km^2^ of core refugia. Only 26.1–27.2% overlapped with existing marine protected areas, leaving 72.8–73.9% unprotected. These findings emphasize the need for climate-informed conservation planning, as most identified potential refugia remain outside the existing MPA network.

## Simple Summary

Many rays are threatened by fishing, habitat degradation, and climate change. We modelled 85 marine ray species and identified coastal and shelf areas likely to remain suitable by 2090–2100. Most lie outside existing marine protected areas, highlighting priorities for conservation and fisheries management.

## 1. Introduction

Marine biodiversity is being threatened by rapid ocean warming and concurrent changes in oxygen availability, ocean chemistry, primary productivity, and the frequency and intensity of extreme climatic events. Distributional shifts have been documented across trophic levels and ocean basins, with many marine taxa tracking changing environmental conditions toward higher latitudes or greater depths [1,2]. Marine heatwaves and cumulative anthropogenic pressures may further accelerate local population declines and alter community composition, particularly in intensively used coastal seas [3,4]. Because most area-based conservation networks were established using historical or contemporary biodiversity patterns, continued climate-driven redistribution may progressively reduce their future ecological representativeness and conservation effectiveness.

Rays are a phylogenetically, morphologically, and ecologically diverse component of marine ecosystems, occupying benthic, demersal, reef-associated, and pelagic habitats and contributing to trophic interactions and ecosystem functioning [5,6]. However, many species are characterized by slow growth, late maturity, low fecundity, long generation times, and limited population recovery [7,8]. These life-history traits increase the conservation importance of habitats that remain suitable under climate change. More than one-third of sharks, rays, and chimeras are threatened with extinction, and substantial losses of abundance, functional diversity, and ecological roles have been documented across oceanic, coastal, and coral-reef ecosystems [8,9,10,11]. Although overfishing remains the dominant direct driver of these declines, climate change may compound the vulnerability of already depleted populations by altering the environmental suitability and spatial configuration of their habitats. Gradual ocean warming and acute thermal anomalies can affect the occurrence, habitat use, physiological performance, locomotor capacity, and dispersal potential of sharks and rays, with responses differing among species according to their mobility, thermal sensitivity, and ecological traits [12,13]. Nevertheless, the global distribution of habitats expected to remain suitable for multiple threatened marine ray species under future climate change remains poorly resolved.

Species distribution models provide a practical approach for evaluating potential habitat redistribution where long-term population monitoring and physiological data are limited [14,15,16]. Comparisons between present-day and future projections can distinguish suitable habitats expected to be lost, retained, or gained, while multispecies stacking can identify areas projected to retain suitable conditions for relatively large numbers of threatened species. Such persistent areas may represent potential climate refugia, broadly defined as locations in which species, populations, or ecological features are comparatively likely to persist under changing climatic conditions [17,18]. Marine climate refugia have been identified using environmental stability, climate velocity, persistence of ecological assemblages, and projected habitat suitability [19,20,21,22]. Climatic persistence alone, however, does not necessarily indicate broader conservation importance or formal protection. Integrating persistent multispecies habitats with complementary conservation-priority layers and the existing marine protected-area network can therefore identify potential refugia with wider conservation relevance while revealing areas that remain outside formal spatial protection [22,23,24].

Here, we projected ensemble species distribution models for 85 threatened marine ray species onto environmental conditions for 2090–2100 under SSP5-8.5 and quantified changes in suitable-habitat richness, including habitat loss, persistence, gain, and net change. We addressed two related questions: where are the principal multispecies climate-resilient hotspots and potential climate refugia for threatened marine rays, and to what extent are these refugia represented within the existing global marine protected-area network? By integrating projected habitat persistence, multi-source conservation-priority evidence, and MPA coverage, this study provides a global spatial basis for identifying climate refugia and protection gaps for threatened marine rays under severe end-of-century climate change.

## 2. Materials and Methods

### 2.1. Study Species and Present-Day Distribution Modeling

Threatened marine ray species were screened from the IUCN Red List of Threatened Species (https://www.iucnredlist.org/, accessed on 18 August 2025). A total of 226 Vulnerable, Endangered, or Critically Endangered ray species with decreasing population trends were initially screened; after excluding 16 primarily freshwater species, 210 marine candidates remained. Occurrence records were compiled from the Global Biodiversity Information Facility (GBIF, https://www.gbif.org/) [25], the Ocean Biodiversity Information System (OBIS, https://obis.org/), and published sources [26,27,28]. Duplicate records, geographically implausible occurrences, and records collected before 1990 were removed. To reduce spatial sampling bias and spatial autocorrelation, occurrence records were spatially thinned using the R (version 4.5.1) package spThin (version 0.2.0), retaining one record per 0.05° grid cell [29]. Following data cleaning and thinning, 85 species with ≥30 post-thinning records were retained for modeling, yielding 30,934 records (Table S1).

Environmental predictors were obtained from Bio-ORACLE (https://bio-oracle.org/) and the Global Marine Environment Datasets (GMED; https://doi.org/10.6084/m9.figshare.5937268, accessed on 15 September 2026) and they were standardized to a global marine grid at a spatial resolution of 0.05° in WGS84 [30]. Separate predictor sets were used for Rajiformes, Torpediniformes, Rhinopristiformes, benthic Myliobatiform rays, and pelagic Myliobatiform rays to account for differences in vertical habitat use and the predictors retained after collinearity filtering (Table 1). Variables with pairwise Pearson correlations of |r| > 0.7 were excluded before model fitting [31].

Ensemble species distribution models were developed in R using the SSDM package (version 0.2.11) [14] and nine algorithms: generalized additive models, generalized linear models, multivariate adaptive regression splines, classification tree analysis, generalized boosted models, maximum entropy models, artificial neural networks, random forests, and support vector machines. Because observed absence data were unavailable, pseudo-absences were generated within the SSDM package using algorithm-specific default strategies based on a recommendation [32]. Models were evaluated using five-fold cross-validation repeated ten times [15,32], and component models with area under the receiver operating characteristic curve (AUC) > 0.9 were retained in the weighted ensembles. Species-level AUC and true skill statistic (TSS) values ranged from 0.937 to 0.993 and from 0.805 to 0.978, respectively (Table S2). Continuous suitability predictions were converted to binary suitable–unsuitable maps using species-specific sensitivity-equals-specificity thresholds [33].

### 2.2. Future Distribution Projections

The fitted ensemble models were projected to Bio-ORACLE environmental conditions for 2090–2100 under SSP5-8.5, based on an ensemble of 11 CMIP6 Earth System Models [34,35]. SSP5-8.5 was selected as a stringent end-of-century stress test for identifying areas retaining suitable conditions under severe climate forcing. For each ecological group, future predictor sets matched the variables and depth strata used in the corresponding present-day models. Climate-dependent predictors retained in each group-specific model were replaced with their corresponding future values, whereas bathymetry, terrain ruggedness, depth, and distance to land were held constant.

Future environmental layers were aligned to the 0.05° WGS84 grid used for present-day modeling and checked for spatial consistency.

The ensemble models were projected onto the future environmental layers using the project function in the SSDM package [14]. The same species-specific thresholds were applied to present-day and future suitability predictions to ensure temporal comparability. The resulting 85 present-day and 85 future binary rasters were matched by species name and verified to have identical spatial properties.

### 2.3. Habitat Change and Climate-Resilient Hotspots

Present-day and future binary suitability maps were compared cell by cell to classify suitable habitat as stable, lost, or gained for each species *i*:

$$Stable_{i}=Current_{i}\times Future_{i}$$

$$Loss_{i}=Current_{i}\times (1-Future_{i})$$

$$Gain_{i}=(1-Current_{i})\times Future_{i}$$

Species-level stable, loss, and gain rasters were summed across the 85 species to derive stable suitable-habitat richness, habitat-loss richness, and habitat-gain richness. Present-day and future binary rasters were also stacked separately to calculate suitable-habitat richness for each period. Net-richness change was calculated as future richness minus present-day richness.

Climate-resilient hotspots were delineated from grid cells with non-zero stable suitable-habitat richness. The 90th-percentile threshold, corresponding to stable richness of at least 36 species, was used to identify the primary Top 10% stable hotspots. The 95th-percentile threshold, corresponding to stable richness of at least 40 species, was used to identify the stricter Top 5% core hotspots. Because stable-richness values were discrete, all cells tied at each percentile threshold were retained. Grid-cell areas were calculated in square kilometers to account for latitudinal variation in geographic cell area. The Top 5% threshold provided a stricter sensitivity test of hotspot delineation and protection-gap estimates (Table S3).

### 2.4. Identification of Potential Ray Climate Refugia

Potential climate refugia were identified by integrating climate-resilient hotspots with three complementary spatial conservation-priority layers: Ecologically or Biologically Significant Marine Areas (https://www.cbd.int/ebsa, accessed on 5 January 2026), Key Biodiversity Areas (https://wdkba.keybiodiversityareas.org/, accessed on 8 January 2026), and ray-specific present-day Zonation priority areas [36,37,38,39,40,41].

The ray-specific priority layer was generated in Zonation 5 using the present-day binary suitability maps of the 85 threatened marine ray species. This layer represented the current conservation value for subsequent assessment of future persistence. Species were weighted according to their IUCN Red List categories, with weights of 5, 4, and 3 assigned to Critically Endangered, Endangered, and Vulnerable species, respectively. Spatial prioritization was conducted using the additive benefit function.

A composite cost layer was constructed from fishing intensity, shipping density, and coastal human disturbance. Fishing and shipping data were obtained from Global Fishing Watch (https://globalfishingwatch.org/, accessed on 10 November 2025) using publicly available 2024 datasets derived from the Automatic Identification System. Activity from vessels classified as trawlers was retained to represent fishing pressure, whereas shipping pressure was represented by AIS-based vessel-density data. Coastal human disturbance was obtained from the corresponding spatial layer provided by Halpern et al. [42]. Fishing intensity and shipping density were transformed using log (x + 1), normalized to a 0–1 range, and resampled to the common 0.05° WGS84 grid. The coastal-disturbance layer was aligned to the same grid, and the three components were combined using equal weights. The highest-ranking 10% of marine grid cells in the resulting Zonation rank map were retained as the ray-specific conservation-priority layer.

The EBSA, KBA, and ray-specific priority layers were converted to binary rasters and aligned to the common 0.05° marine grid. Following an adapted conservative spatial-agreement framework [22], each grid cell received one point for overlap with each layer. Cells supported by at least two of the three layers were classified as multi-source conservation-priority areas (Figure S1).

Potential climate refugia were defined as areas where climate-resilient hotspots overlapped with multi-source conservation-priority areas. The Top 10% stable hotspots were used to delineate the primary refugia layer, whereas the Top 5% stable hotspots were used to identify the stricter core-refugia layer.

### 2.5. Assessment of MPA Coverage and Protection Gaps

Marine protected-area boundaries were obtained from the World Database on Protected Areas through Protected Planet (https://www.protectedplanet.net/en, accessed on 5 January 2026) [43]. MPA polygons were converted to a binary raster aligned to the common 0.05° WGS84 marine grid.

Potential-refugia cells overlapping with existing MPAs were classified as MPA-covered refugia, whereas refugia outside MPAs were classified as unprotected refugia. For both the Top 10% and Top 5% hotspot thresholds, total refugia area, MPA-covered area, unprotected area, MPA coverage percentage, and protection-gap percentage were calculated. The Top 10% threshold was used for the primary analysis, whereas the Top 5% threshold provided a stricter sensitivity assessment.

## 3. Results

### 3.1. Present-Day and Future Species Richness

**Figure 1:**
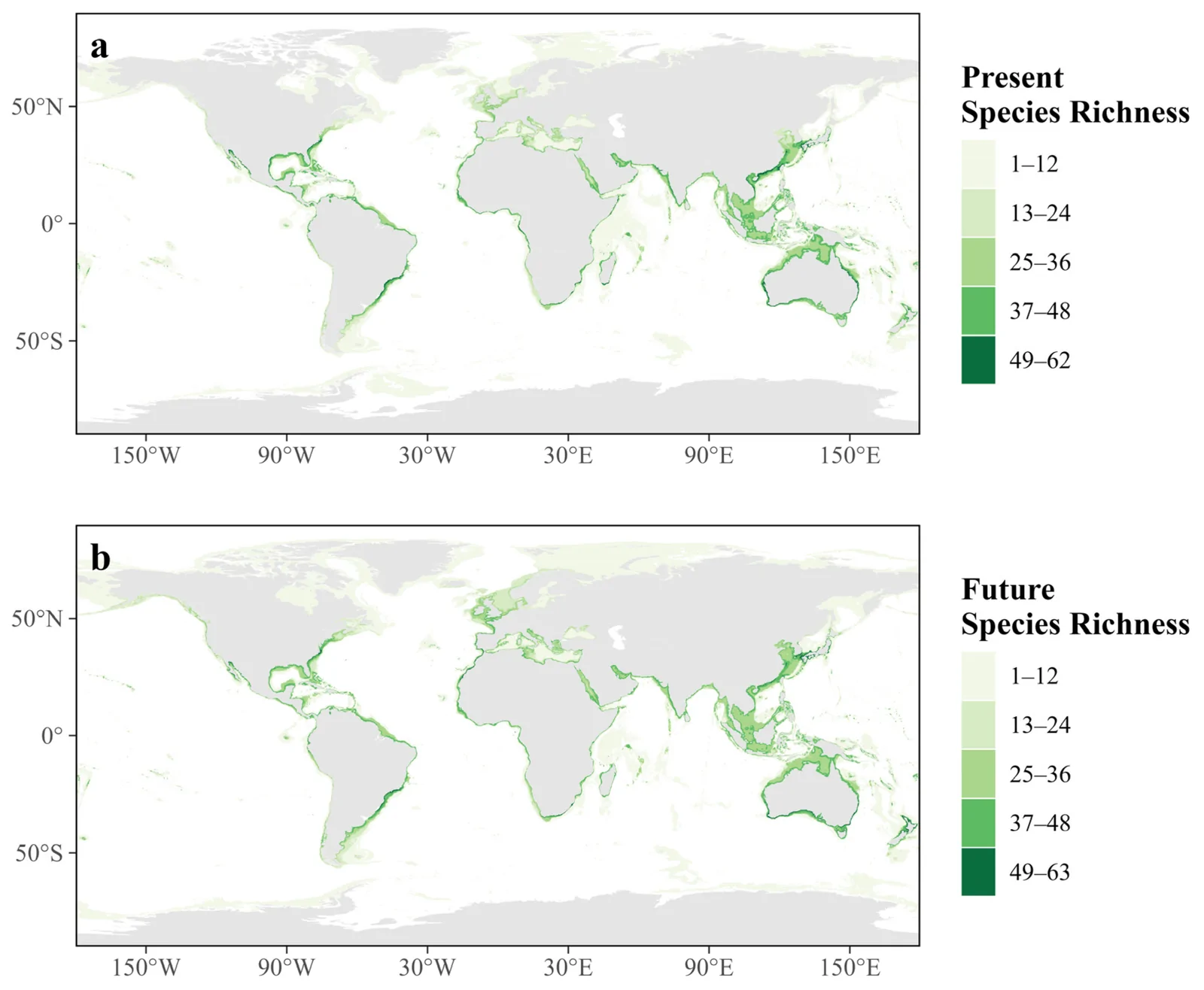
Present-day and projected future suitable-habitat richness of 85 threatened marine ray species: (**a**) present-day suitable-habitat richness; (**b**) projected suitable-habitat richness under SSP5-8.5 for 2090–2100.

Present-day suitable-habitat richness reached a maximum of 62 species, whereas projected future richness reached a maximum of 63 species under SSP5-8.5 for 2090–2100 (Figure 1). Under both present-day and future conditions, suitable-habitat richness was concentrated predominantly in coastal and continental-shelf waters. Major concentrations occurred in the Indo-West Pacific, the Caribbean Sea and Gulf of Mexico, the Mediterranean Sea and northeastern Atlantic, East Asian marginal seas, and the coastal waters of Australia.

Although the broad global distribution of suitable-habitat richness was retained under future conditions, regional differences were evident between the two periods. Future richness was locally lower across several tropical and subtropical coastal regions, whereas higher future richness was more apparent at higher latitudes, particularly in the northern North Atlantic, northern North Pacific, and waters surrounding southern South America.

### 3.2. Projected Changes in Suitable-Habitat Richness

**Figure 2:**
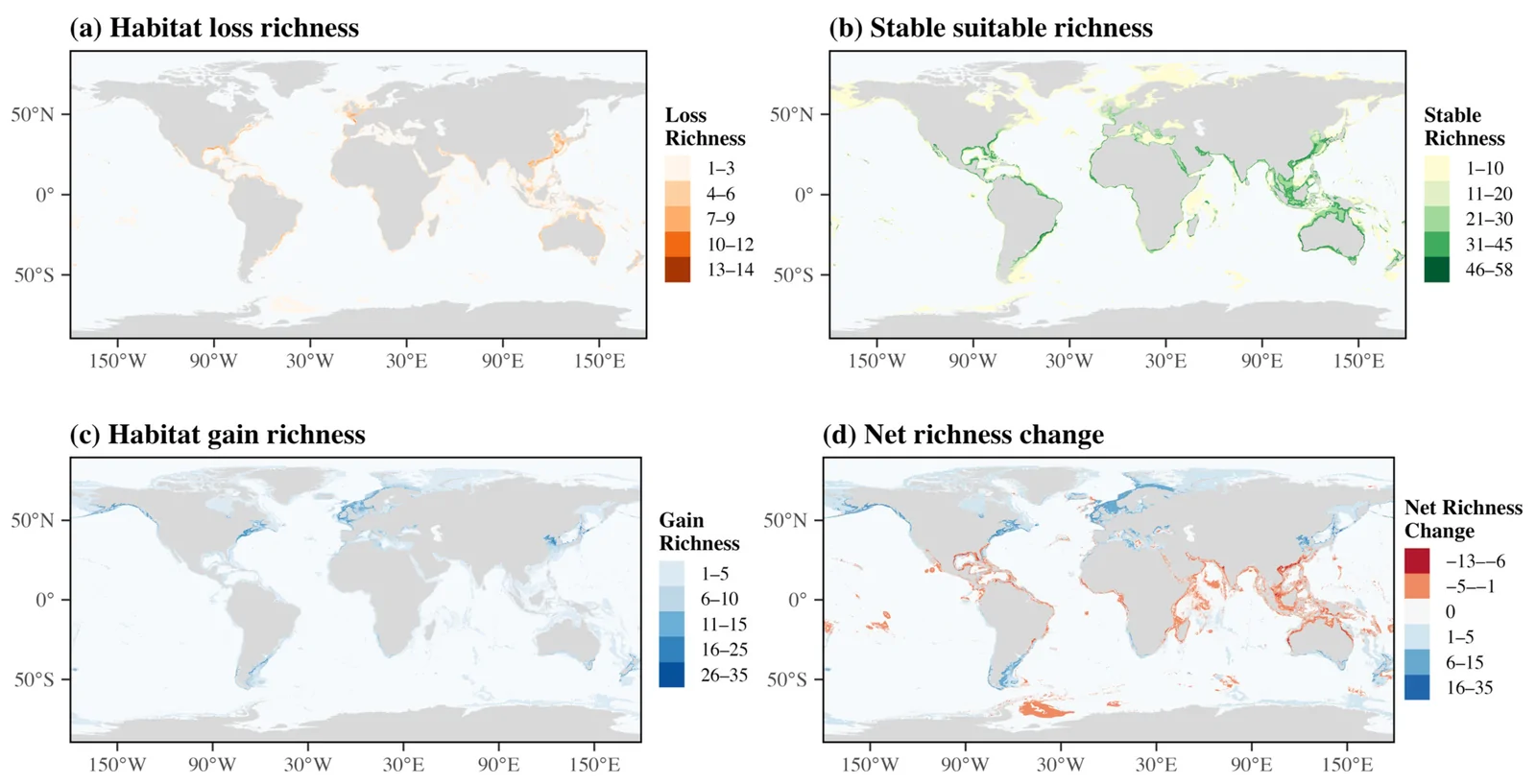
Projected changes in suitable-habitat richness of threatened marine rays under SSP5-8.5 for 2090–2100: (**a**) habitat-loss richness; (**b**) stable suitable-habitat richness; (**c**) habitat-gain richness; (**d**) net-richness change, calculated as future richness minus present-day richness.

Projected changes in suitable-habitat richness showed marked spatial heterogeneity under SSP5-8.5 for 2090–2100 (Figure 2). Stable suitable-habitat richness reached a maximum of 58 species, whereas habitat-loss and habitat-gain richness reached maxima of 14 and 35 species, respectively.

Stable suitable-habitat richness was concentrated mainly in coastal and continental-shelf waters, with prominent concentrations in the Indo-West Pacific, the Caribbean Sea and Gulf of Mexico, the Mediterranean Sea and northeastern Atlantic, East Asian marginal seas, and coastal Australia. Habitat loss occurred widely along tropical and subtropical coastal waters, particularly in parts of the Indo-West Pacific, the Caribbean region, and other intensively occupied shelf systems. By contrast, habitat gain was more extensive at higher latitudes, especially in the northern North Atlantic, northern North Pacific, and waters around southern South America.

Net-richness change, calculated as future richness minus present-day richness, ranged from −13 to 35 species. Negative values indicated local net reductions in the number of species for which suitable habitat was projected, rather than negative species richness, whereas positive values indicated local net gains. Negative net changes occurred mainly in tropical and subtropical coastal regions, while positive net changes were concentrated predominantly in higher-latitude waters.

### 3.3. Climate-Resilient Hotspots

**Figure 3:**
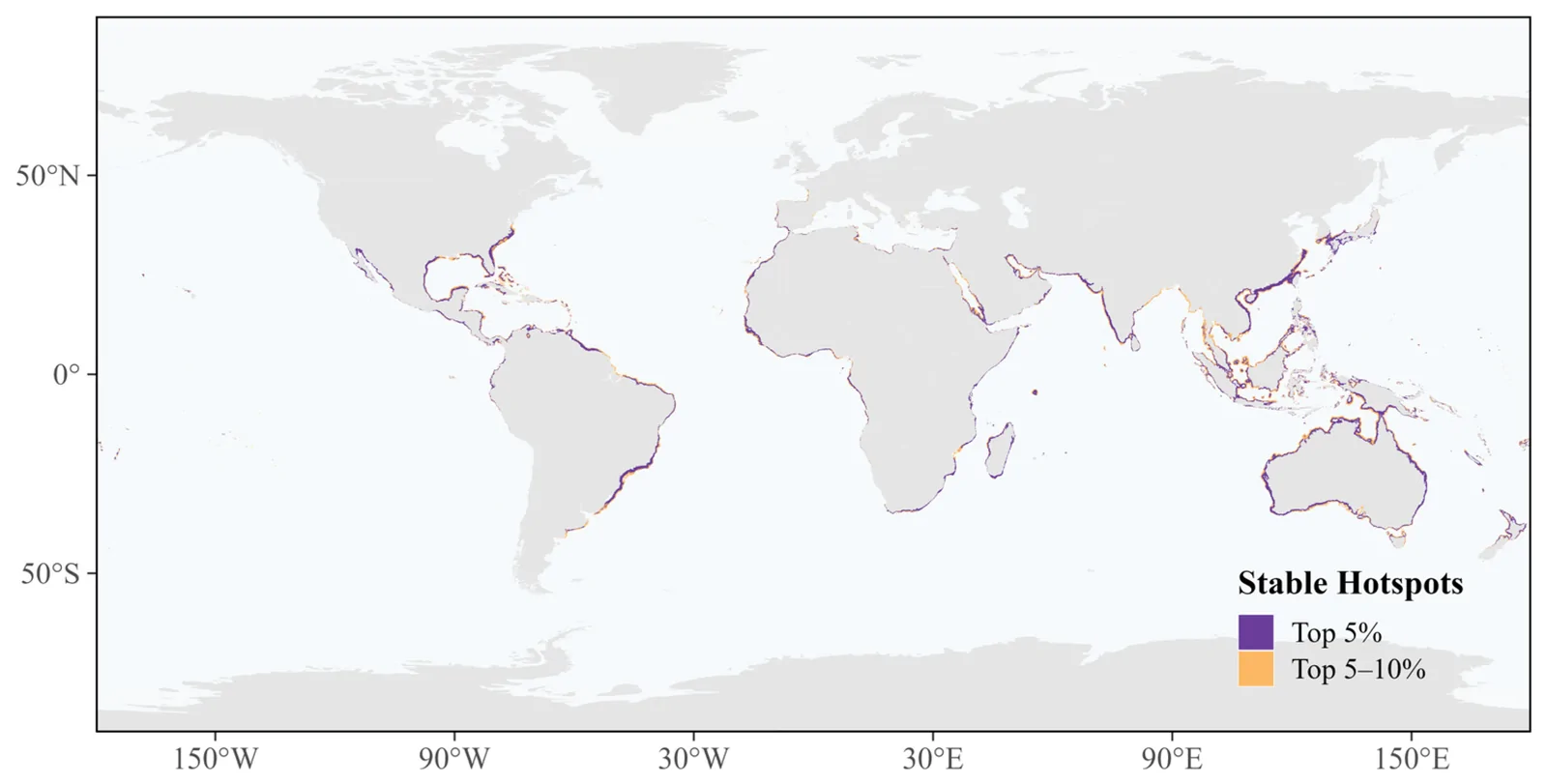
Global distribution of climate-resilient hotspots for threatened marine rays under SSP5-8.5 for 2090–2100. The Top 10% stable hotspots comprise the Top 5% core hotspots and the Top 5–10% hotspot zone, delineated using the 95th- and 90th-percentile thresholds of stable suitable-habitat richness, respectively.

Areas retaining suitable habitats for at least one threatened marine ray species covered approximately 49.15 million km^2^. Stable suitable-habitat richness was highest along continental shelves, marginal seas, and archipelagic waters, with major concentrations in the Indo-West Pacific, the Caribbean Sea and Gulf of Mexico, the Mediterranean Sea and northeastern Atlantic, East Asian marginal seas, and coastal Australia (Figure 3).

The 90th-percentile threshold corresponded to stable richness of at least 36 species and delineated approximately 6.47 million km^2^ of Top 10% stable hotspots. The 95th-percentile threshold corresponded to stable richness of at least 40 species and delineated approximately 3.89 million km^2^ of Top 5% core hotspots.

Because stable-richness values were discrete and all cells tied at each percentile threshold were retained, the delineated Top 10% and Top 5% hotspot areas represented 13.17% and 7.91%, respectively, of the total area with non-zero stable suitable-habitat richness. The Top 5% core hotspots formed a spatial subset of the broader Top 10% stable-hotspot distribution.

### 3.4. Potential Ray Climate Refugia

Multi-source conservation-priority areas, defined as cells supported by at least two out of three complementary conservation-priority layers, covered approximately 2.88 million km^2^. These areas were distributed mainly across coastal waters, continental shelves, marginal seas, and archipelagic regions.

**Figure 4:**
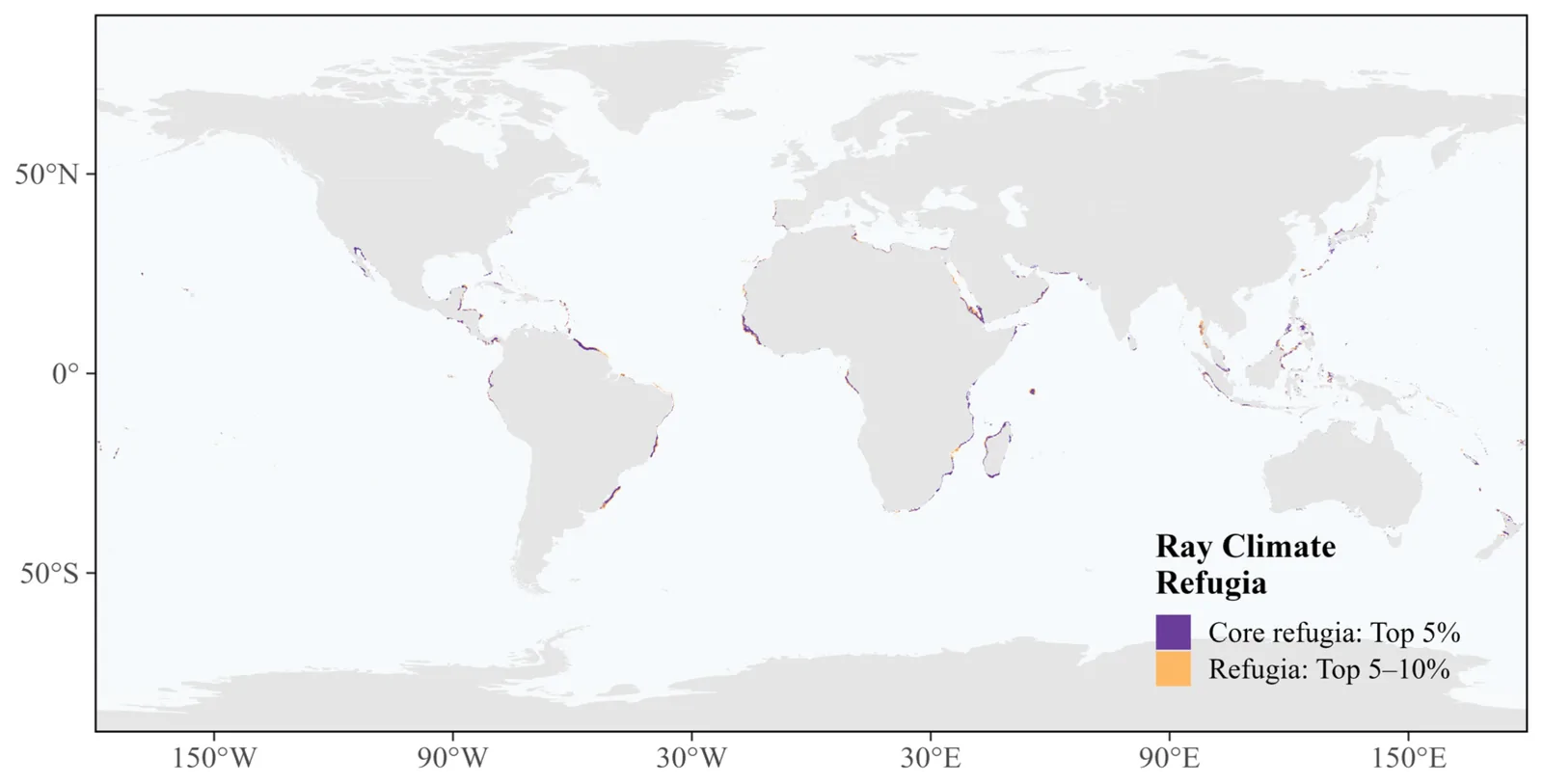
Global distribution of potential climate refugia for threatened marine rays. Potential refugia were identified by intersecting climate-resilient hotspots with multi-source conservation-priority areas supported by at least two of three complementary spatial layers. The Top 10% refugia comprise the Top 5% core refugia and the Top 5–10% refugia zone.

Intersecting the multi-source conservation-priority areas with the Top 10% stable hotspots identified approximately 1.64 million km^2^ of potential climate refugia, equivalent to 25.27% of the corresponding stable-hotspot area (Figure 4). Under the stricter threshold, intersecting these areas with the Top 5% stable hotspots identified approximately 1.06 million km^2^ of core refugia, representing 27.20% of the Top 5% hotspot area.

Potential climate refugia were concentrated mainly in parts of the Indo-West Pacific, the Caribbean Sea and Gulf of Mexico, the Mediterranean Sea and northeastern Atlantic, East Asian marginal seas, coastal Australia, and several sections of the eastern Pacific and western Indian Ocean. The Top 5% core refugia were nested within the broader Top 10% refugia distribution.

### 3.5. MPA Coverage and Protection Gaps of Potential Climate Refugia

**Figure 5:**
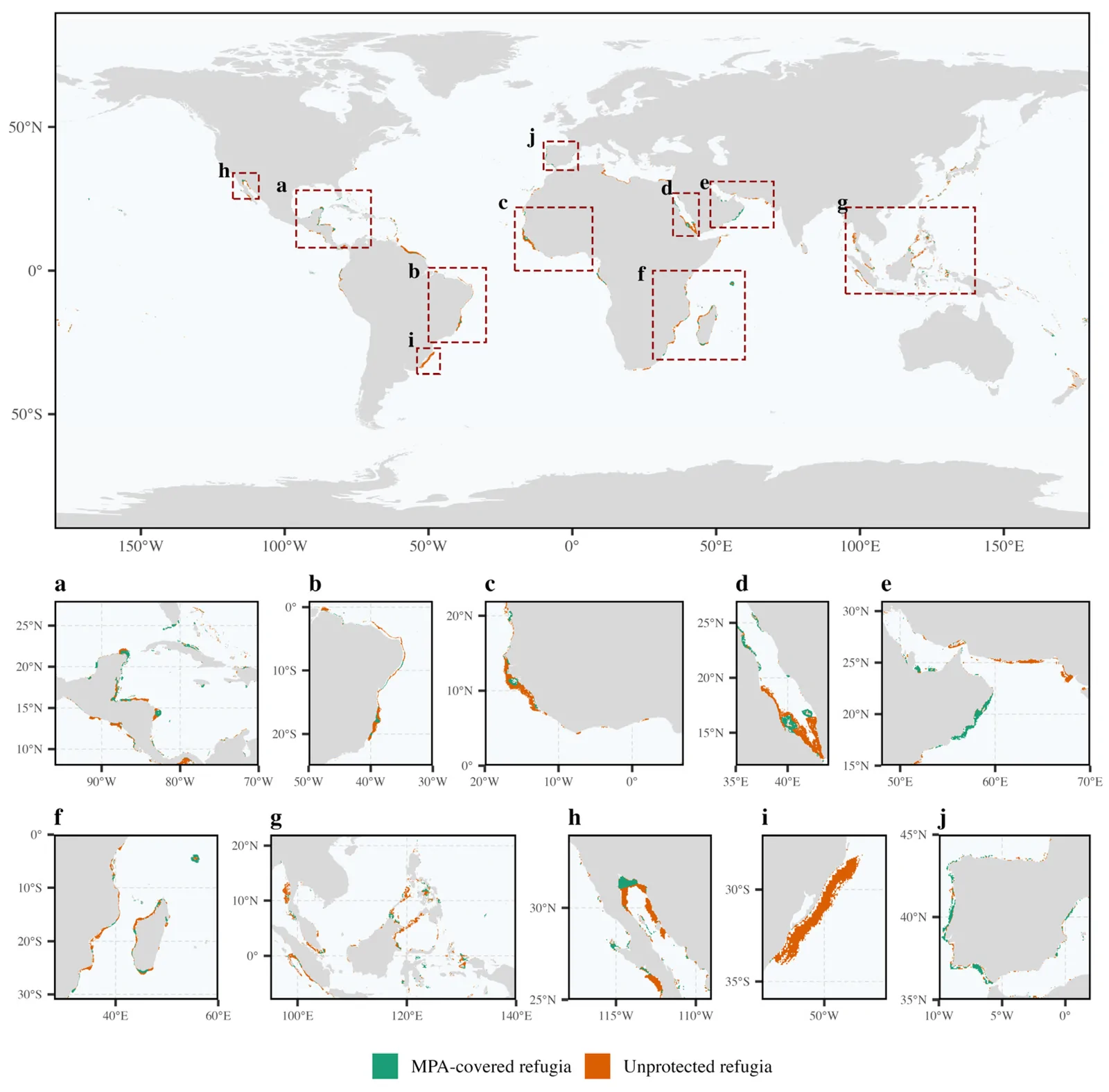
Marine protected-area coverage and protection gaps of potential climate refugia for threatened marine rays. Refugia overlapping with existing marine protected areas are classified as MPA-covered refugia, whereas refugia outside the protected-area network are classified as unprotected refugia. Enlarged regional views show (**a**) the Caribbean Sea, Gulf of Mexico, and Central America; (**b**) the western South Atlantic off Brazil; (**c**) the Gulf of Guinea and the eastern tropical Atlantic off West Africa; (**d**) the Red Sea and the Bab el-Mandeb–western Gulf of Aden region; (**e**) the Persian Gulf, Gulf of Oman, and northwestern Arabian Sea; (**f**) the Mozambique Channel, Madagascar, and the adjacent southwestern Indian Ocean; (**g**) Southeast Asian seas and the adjacent western Pacific; (**h**) Baja California and the Gulf of California; (**i**) the southern Brazil–Uruguay shelf in the southwestern Atlantic; (**j**) the Iberian Atlantic margin, Strait of Gibraltar, and western Mediterranean.

Existing marine protected areas covered approximately 427,396 km^2^ of the potential refugia identified using the Top 10% stable-hotspot threshold, accounting for 26.12% of the total refugia area (Figure 5). The remaining 1,208,649 km^2^, corresponding to 73.88%, occurred outside the existing MPA network and were classified as protection gaps.

For the Top 5% core-refugia layer, approximately 287,344 km^2^ overlapped existing MPAs, representing 27.17% of the total core-refugia area. The remaining 770,157 km^2^, or 72.83%, occurred outside MPAs. MPA coverage differed by only 1.05 percentage points between the two hotspot thresholds, indicating that the conclusion of limited protection was consistent under both hotspot definitions.

MPA-covered refugia were generally fragmented and spatially limited, whereas unprotected refugia were more extensive across coastal waters, continental shelves, and island-associated seas. Protected and unprotected refugia were concentrated across multiple coastal regions spanning the Atlantic, Indian, and Pacific Oceans (Figure 5).

## 4. Discussion

Our results reveal a marked spatial redistribution of suitable habitats for threatened marine rays under SSP5-8.5 by 2090–2100. Habitat-loss and negative net-richness changes were concentrated mainly in tropical and subtropical coastal waters, whereas habitat gains and positive net changes occurred more extensively at higher latitudes. Despite this redistribution, extensive suitable habitats were projected to persist along continental shelves, marginal seas, and archipelagic waters. However, only approximately one-quarter of the most species-rich stable hotspots coincided with multi-source conservation-priority areas, and less than 28% of the resulting potential climate refugia were covered by existing MPAs. These results indicate that future habitat persistence and present-day spatial protection remain poorly aligned for threatened marine rays.

### 4.1. Redistribution and Persistence of Suitable Habitats

The broad contrast between projected habitat losses at lower latitudes and gains at higher latitudes is consistent with the poleward redistribution documented for many marine taxa under ocean warming [1,44,45]. Marine organisms frequently track spatial changes under environmental conditions, although the rate and direction of redistribution vary among regions because climate velocity, oceanography, coastline configuration, and species-specific thermal affinities and ecological constraints differ spatially [44,45,46]. Marine heatwaves and other extreme events may further alter local habitat suitability beyond changes associated with long-term mean conditions [3]. Accordingly, the patterns identified here should be interpreted as changes in potential environmental suitability rather than uniform poleward range shifts.

The concentration of stable suitable-habitat richness in coastal and continental-shelf waters reflects the strong habitat association of many threatened rays. These environments support high marine biodiversity but are also exposed to fisheries, coastal development, shipping, pollution, and habitat modification [7,8,47]. For rays, continued suitability in these areas does not necessarily imply low overall vulnerability. Slow growth, delayed maturity, low fecundity, and limited population recovery can amplify the effects of fishing mortality and habitat degradation even where environmental conditions remain suitable [7,8]. Stable suitable-habitat hotspots therefore represent areas of projected climatic persistence, rather than areas free from anthropogenic pressure.

The persistence of high richness in the Indo-West Pacific, the Caribbean Sea–Gulf of Mexico, the Mediterranean and northeastern Atlantic, East Asian marginal seas, and coastal Australia indicates that several present-day centers of threatened-ray diversity may retain substantial conservation value by the end of the century. The Indo-West Pacific is particularly notable because its extensive shelves, archipelagic systems, and high habitat heterogeneity support globally important concentrations of marine biodiversity [47]. Nevertheless, the coexistence of persistent suitability and projected local losses within these regions indicates that their species composition may change even where total richness remains relatively high.

### 4.2. Climate-Resilient Hotspots and Potential Ray Climate Refugia

Climate-change refugia are generally regarded as areas capable of supporting the persistence of species, populations, or ecological communities under changing climatic conditions [17,48]. In this study, climate-resilient hotspots were identified from stable suitable-habitat richness rather than from the stability of a single environmental variable. This multispecies approach emphasized areas projected to retain suitable conditions for relatively large numbers of threatened rays and therefore provided a taxon-specific measure of refugial capacity.

However, climatic persistence alone does not establish conservation priority. By requiring stable hotspots to overlap with multi-source conservation-priority areas, the refugia framework retained only locations supported by at least two complementary sources of conservation evidence. Approximately 25.27% of the Top 10% stable-hotspot area and 27.20% of the Top 5% hotspot area met this criterion. The limited overlap shows that many climatically persistent habitats were not simultaneously recognized by the EBSA, KBA, and ray-specific Zonation layers. Conversely, conservation-priority areas that did not overlap with stable hotspots may not retain the same multispecies suitability under severe end-of-century climate conditions.

The general framework is consistent with recent global marine-refugia research that combines projected climate stability with agreement among multiple conservation-priority layers [22]. The potential refugia identified here covered a much smaller area than the global marine climate refugia reported by that study, which is expected because the present analysis was restricted to 85 threatened ray species and used a ray-specific stable-richness threshold and conservation-priority layer. The similar proportions of stable hotspots retained as refugia under the Top 10% and Top 5% thresholds suggest that the broad spatial relationship between climatic persistence and multi-source conservation-priority evidence was not highly sensitive to hotspot definition.

Potential refugia remained concentrated mainly in coastal and shelf waters rather than forming extensive offshore areas. This pattern reflects the spatial ecology of the focal species and highlights the need to interpret climate refugia in relation to taxonomic and ecological contexts. For threatened rays, refugia should therefore be viewed as areas with comparatively high potential to retain multispecies-suitable habitats, not as permanently unchanged environments or guaranteed sites of long-term population persistence.

### 4.3. Conservation Implications of MPA Coverage and Protection Gaps

Existing MPAs covered only 26.12% of the refugia identified under the primary threshold and 27.17% under the stricter threshold. The consistency of these estimates indicates that the conclusion of limited MPA representation was robust to the choice of stable-hotspot threshold. More than 72% of potential refugia remained outside the existing MPA network under both definitions, revealing a substantial spatial mismatch between projected climate persistence and the current formal protection.

Well-designed and effectively managed marine reserves can reduce local pressures and strengthen ecological resilience to climate change [49]. Nevertheless, MPA designation alone does not guarantee effective conservation of sharks and rays. Outcomes depend on the spatial correspondence between protected boundaries and critical habitats, the level of fishing restriction, enforcement, life-stage coverage, movement behavior, and the extent of surrounding fisheries’ mortality [50,51]. This distinction is particularly important for rays because many species occupy shallow coastal waters where fishing efforts and other human activities are concentrated [7,8].

Major concentrations of unprotected refugia occurred in the Caribbean and Central America, the western tropical South Atlantic, the Gulf of Guinea and adjacent West Africa, the Red Sea–Arabian Sea, the southwestern Indian Ocean, and Southeast Asia. These regions differ substantially in governance capacity, fisheries structure, habitat conditions, and existing protected-area coverage. The spatial results should therefore not be interpreted as direct proposals for new MPA boundaries. Instead, they identify broad areas in which finer-scale ecological validation and management assessment should be prioritized.

In areas where new or expanded MPAs are feasible, potential refugia could inform the placement of climate-resilient protected areas. In heavily used coastal waters, complementary measures may be equally important, including OECMs, seasonal or gear-based fishery restrictions, bycatch mitigation, protection of nursery and breeding habitats, and coordination across jurisdictional boundaries. Given the severe global decline in sharks and rays under sustained fishing pressure, reducing mortality within and around refugial habitats will remain necessary even where future environmental suitability is relatively stable [8,9].

### 4.4. Limitations and Conservation Implications

Several limitations should be considered when interpreting these results. First, the future projections were based on correlative species distribution models that assume that present-day species–environment relationships remain transferable to future conditions. Novel environmental combinations, physiological adaptation, biotic interactions, seasonal and vertical movements, demographic processes, and evolutionary responses were not explicitly represented. Spatially blocked cross-validation was not performed; therefore, model evaluation may remain influenced by spatial autocorrelation. Second, no dispersal constraints were imposed. Projected habitat gains indicate newly suitable environmental conditions rather than a realized range expansion.

Third, the analysis focused on SSP5-8.5 and thus represents habitat persistence under severe end-of-century climate forcing rather than the full range of possible climate futures. Static predictors, including bathymetry, terrain ruggedness, and distance to land, were held constant, while changes in habitat structure, coastal development, and future fishing pressures were not modeled. Uneven occurrence-data availability may affect model performance and limit the representation of data-poor species.

Finally, MPA overlap measured spatial representation but not management effectiveness, enforcement, or protection level. Similarly, EBSAs and KBAs indicate ecological importance but do not confer legal protection. The identified refugia should therefore be regarded as priorities for subsequent assessment rather than definitive management units. Site-level validation using fisheries observations, tracking data, population monitoring, and regional environmental projections will be required before specific conservation boundaries or regulations are proposed.

Despite these limitations, the analysis provides a spatially explicit framework linking future habitat persistence, multi-source conservation-priority evidence, and formal protection. The large proportion of potential ray climate refugia outside existing MPAs indicates that conservation planning based solely on present-day distributions may overlook habitats with long-term value. Incorporating projected persistence into protected-area planning and fisheries management could improve the capacity of marine conservation networks to retain threatened-ray biodiversity under continued ocean change.

## 5. Conclusions

Climate change is likely to alter the spatial distribution of suitable habitats for threatened marine rays, potentially reducing the long-term representativeness of existing conservation networks. By integrating projected habitat persistence with complementary conservation-priority layers, this study identifies potential climate refugia and shows that most remain outside current MPAs. These findings provide a spatial basis for incorporating climate persistence into marine biodiversity conservation, while highlighting the need for finer-scale validation and complementary fisheries and area-based management.

## Figures and Tables

**Table 1:** Environmental predictors retained for species distribution modeling and future projection.

Variable	Unit	Spatial Resolution	Source
Temperature	°C	0.05°	Bio-ORACLE
Salinity	-	0.05°	Bio-ORACLE
Velocity	m·s^−1^	0.05°	Bio-ORACLE
Phytoplankton concentration	mmol·m^−3^	0.05°	Bio-ORACLE
Dissolved oxygen	mmol·m^−3^	0.05°	Bio-ORACLE
Chlorophyll-*a*	mg·m^−3^	0.05°	Bio-ORACLE
Nitrate	mmol·m^−3^	0.05°	Bio-ORACLE
Phosphate	mmol·m^−3^	0.05°	Bio-ORACLE
Terrain ruggedness index	-	0.05°	Bio-ORACLE
Bathymetry	m	0.05°	Bio-ORACLE
Depth	m	0.083°	GMED
Distance to land	km	0.083°	GMED

Group-specific combinations of predictors were retained after collinearity filtering. During future projection, climate-dependent predictors were replaced with their corresponding SSP5-8.5 values for 2090–2100, whereas terrain ruggedness index, bathymetry, depth, and distance to land were held constant at present-day values when included in the corresponding models. All predictor layers were resampled and aligned to a common 0.05° WGS84 analysis grid before model fitting and projection.

## Data Availability

The dataset supporting the findings of this research is available upon request.

## Supplementary Materials

The following supporting information can be downloaded at: https://www.mdpi.com/article/10.3390/ani16182924/s1. Figure S1. Spatial construction of multi-source conservation-priority areas used to identify potential climate refugia. (a) Ecologically or Biologically Significant Marine Areas (EBSAs); (b) Key Biodiversity Areas (KBAs); (c) present-day Top 10% Zonation priorities for threatened marine rays; and (d) multi-source conservation-priority areas supported by at least two of the three spatial layers. Light purple indicates cells supported by two layers, whereas dark purple indicates cells supported by all three layers. Figure S2. Species-level future binary suitable-habitat projections for 85 threatened marine ray species under SSP5-8.5 for 2090–2100. Green cells indicate areas classified as suitable using species-specific sensitivity-equals-specificity thresholds. Table S1. Threatened marine ray species included in the modelling analysis and occurrence-data summary. Table S2. Summary of ensemble model performance across five groups of threatened marine rays. Table S3. Sensitivity analysis under the Top 10% and Top 5% stable-hotspot thresholds.
