# Supplementary material for: Global Potential Refugia and Conservation Gaps for Threatened Marine Rays Under Future Climate Change
Source: Animals (Basel). 2026 Sep 17;16(18):2924. doi: 10.3390/ani16182924 (PMC13603717; doi:10.3390/ani16182924)
Supplement: Supplementary file 1 [file animals-16-02924-s001.zip › Figure_S1_multi-source conservation-priority areas_construction.pdf]

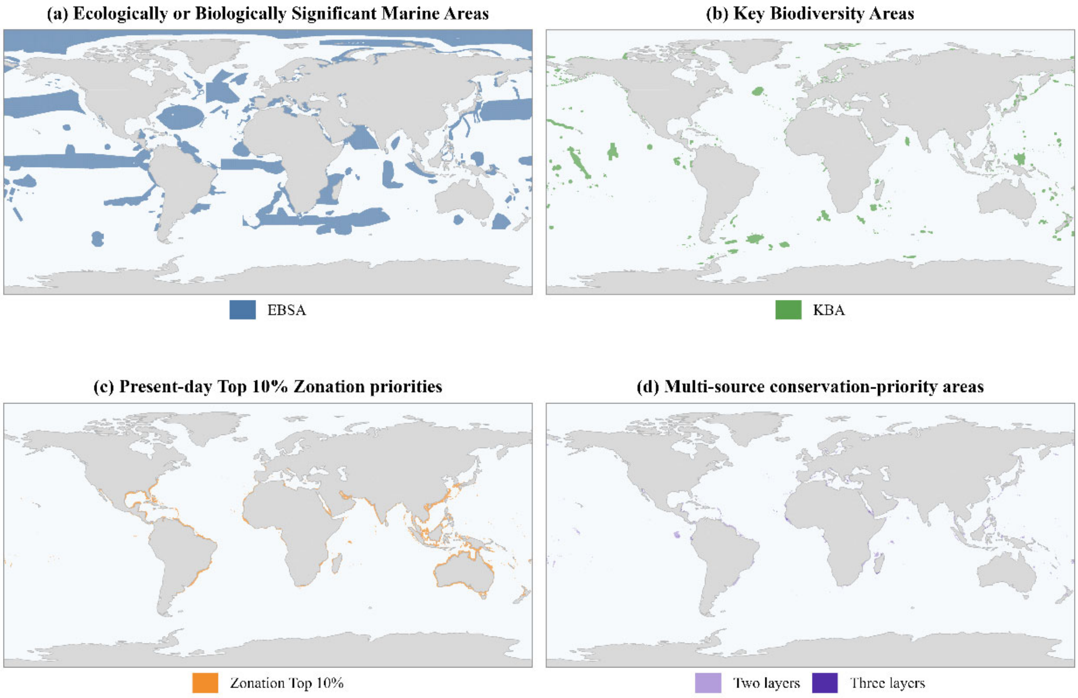

Figure S1. Spatial construction of multi-source conservation-priority areas used to identify potential climate refugia. (a) Ecologically or Biologically Significant Marine Areas (EBSAs); (b) Key Biodiversity Areas (KBAs); (c) present-day Top 10% Zonation priorities for threatened marine rays; and (d) multi-source conservation-priority areas supported by at least two of the three spatial layers. Light purple indicates cells supported by two layers, whereas dark purple indicates cells supported by all three layers.
