# Supplementary material for: Global Potential Refugia and Conservation Gaps for Threatened Marine Rays Under Future Climate Change
Source: Animals (Basel). 2026 Sep 17;16(18):2924. doi: 10.3390/ani16182924 (PMC13603717; doi:10.3390/ani16182924)

Supplementary Figure S2

Global Potential Refugia and Conservation Gaps for Threatened Marine Rays under Future Climate Change

Caption: Species-level future binary suitable-habitat projections for 85 threatened marine ray species under SSP5-8.5 for 2090–2100. Green cells indicate areas classified as suitable using species-specific sensitivity-equals-specificity thresholds.

Supplementary Figures

Future Binary Suitable-Habitat Maps for 85 Threatened Marine Ray Species

---

This supplementary atlas contains species-level future binary suitable-habitat maps under SSP5-8.5 for 2090–2100. Species are organized into five analytical groups consistent with the manuscript. Each figure page contains six species maps arranged in three columns by two rows.

Figure index

Figure S2(a). Future binary suitable-habitat projections for Rajiformes under SSP5-8.5 for 2090–2100 (24 species; pages 1-4).

Figure S2(b). Future binary suitable-habitat projections for Rhinopristiformes under SSP5-8.5 for 2090–2100 (14 species; pages 5-7).

Figure S2(c). Future binary suitable-habitat projections for Torpediniformes under SSP5-8.5 for 2090–2100 (6 species; page 8).

Figure S2(d). Future binary suitable-habitat projections for benthic Myliobatiformes under SSP5-8.5 for 2090–2100 (23 species; pages 9-12).

Figure S2(e). Future binary suitable-habitat projections for pelagic Myliobatiformes under SSP5-8.5 for 2090–2100 (18 species; pages 13-15).

Figure S2(a). Future binary suitable-habitat projections for Rajiformes.

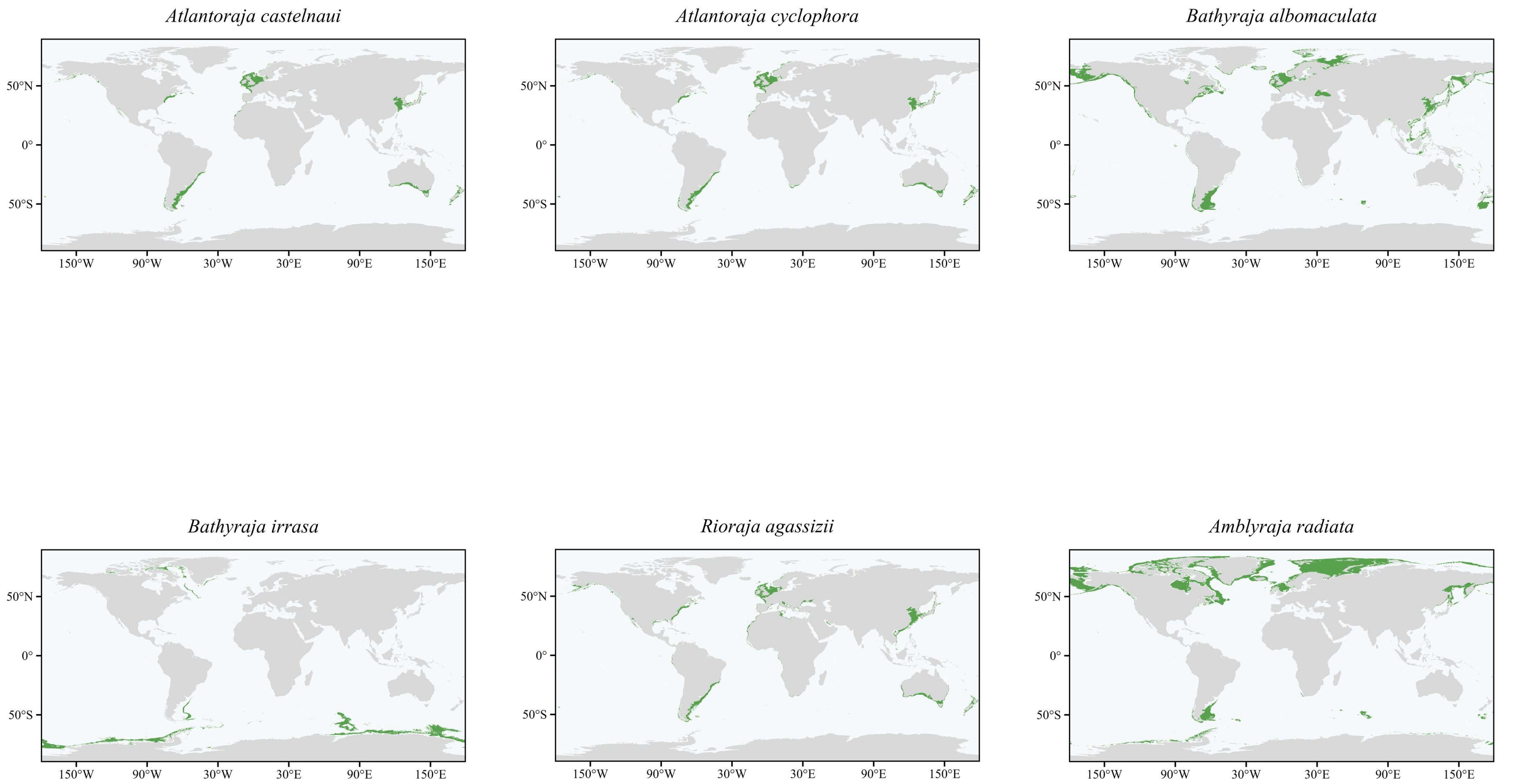

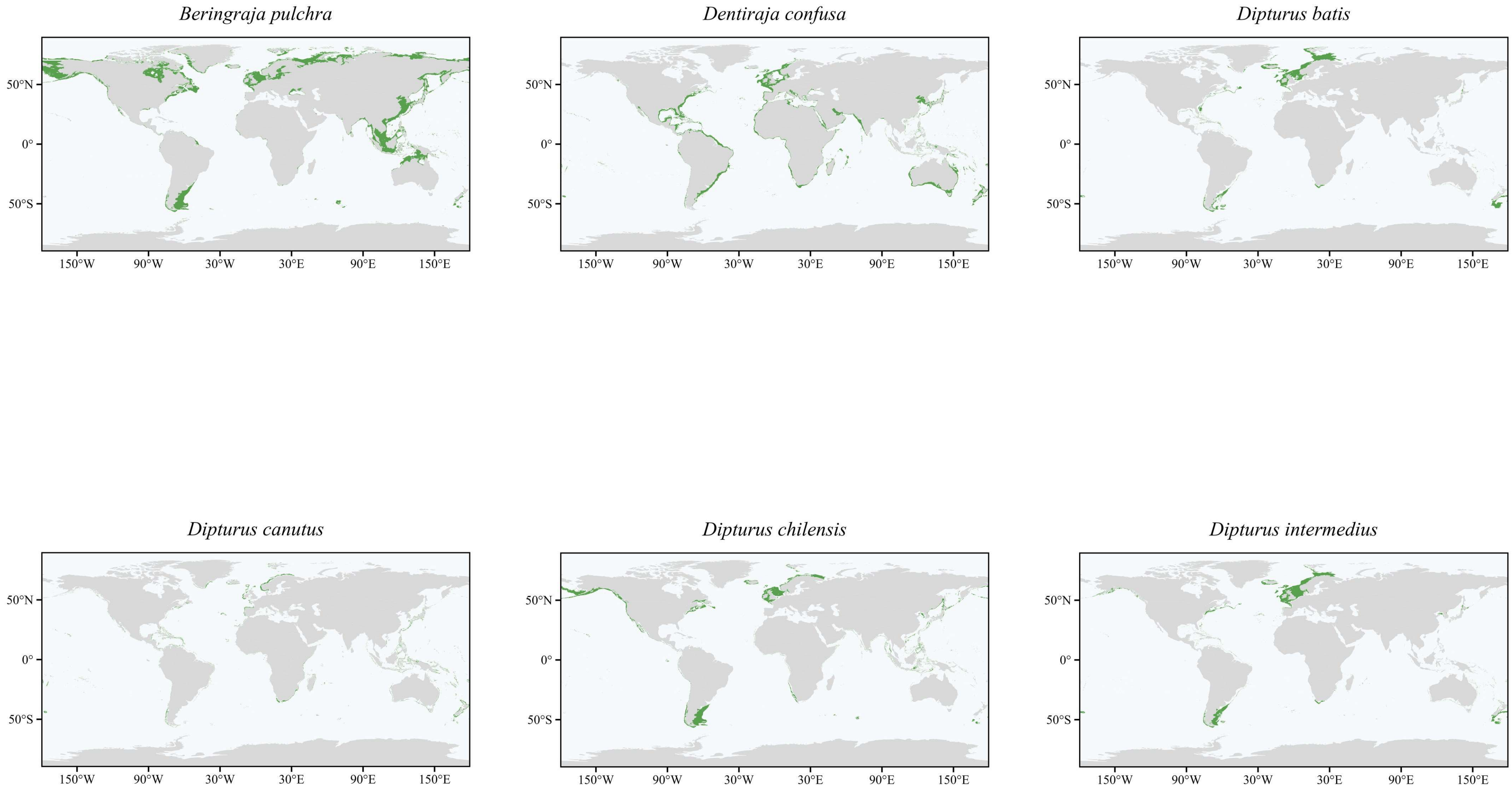
Beringraja pulchra
Dentiraja confusa
Dipturus batis
Dipturus canutus
Dipturus chilensis
Dipturus intermedius
50°N
0°
50°S
150°W
90°W
30°W
30°E
90°E
150°E

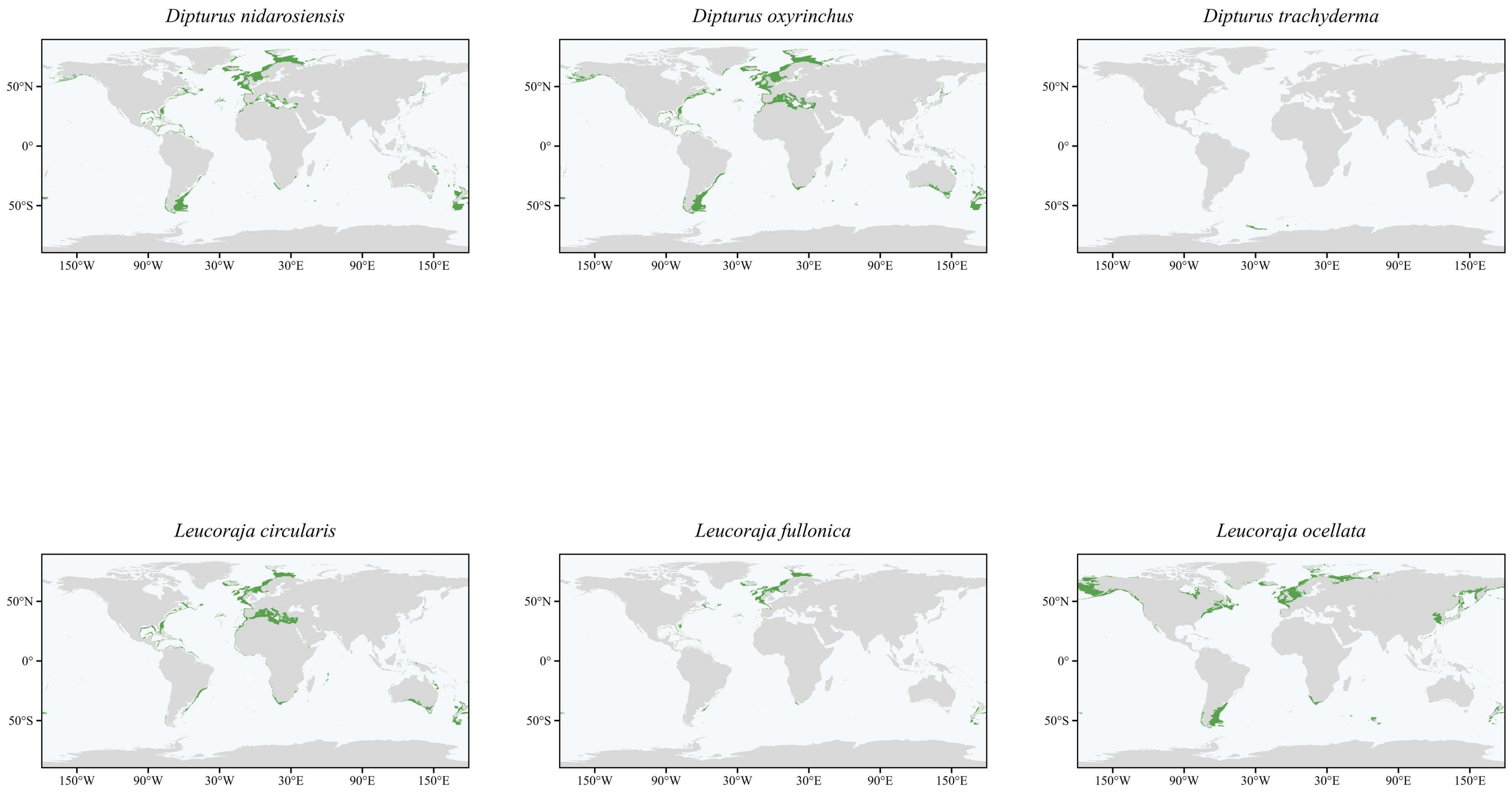
Dipturus nidarosiensis
Dipturus oxyrinchus
Dipturus trachyderma
Leucoraja circularis
Leucoraja fullonica
Leucoraja ocellata
50°N
0°
50°S
150°W
90°W
30°W
30°E
90°E
150°E

Leucoraja wallacei

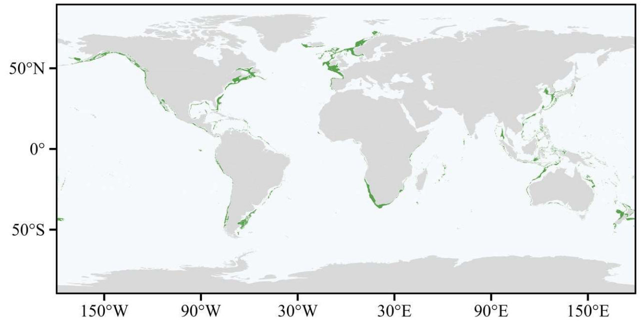

Okamejei acutispina

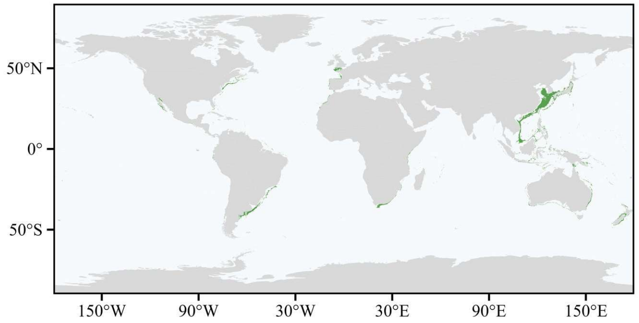

Okamejei kenojei

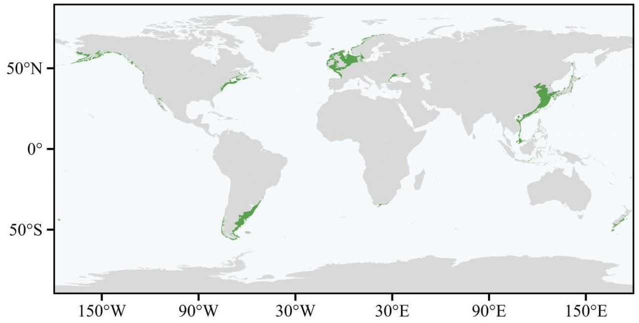

Raja radula

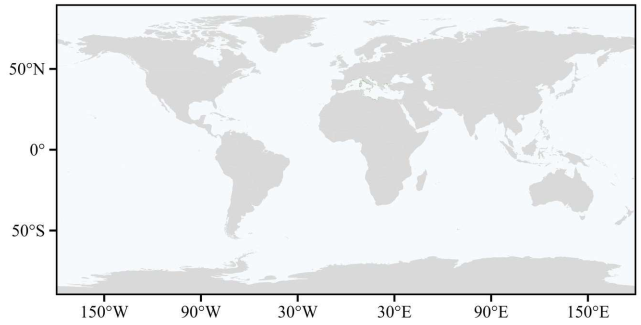

Rostroraja alba

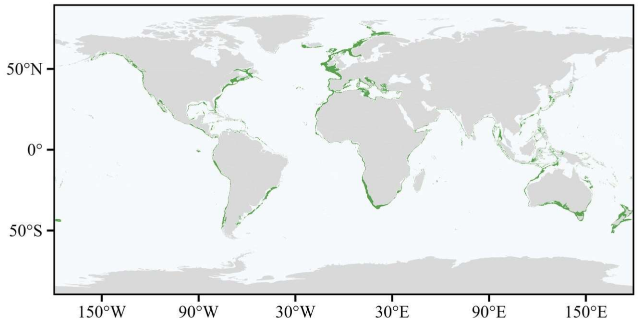

Spiniraja whitleyi

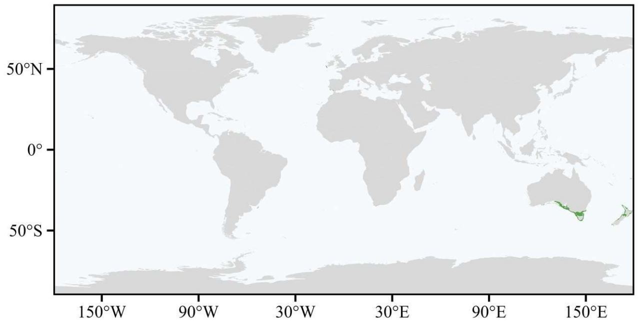

Acroteriobatus annulatus

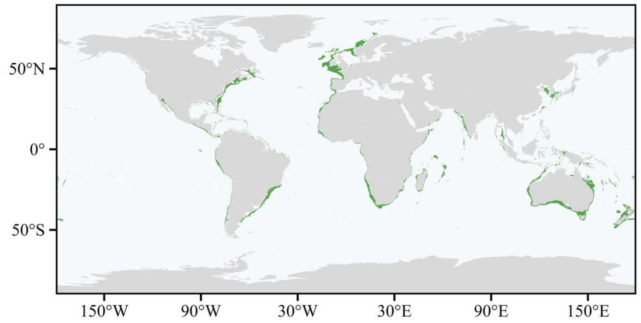

Pseudobatos horkelii

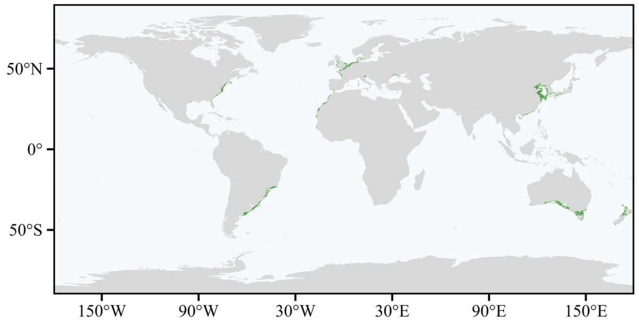

Pseudobatos lentiginosus

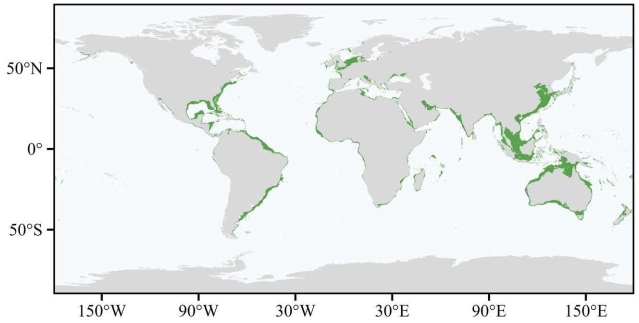

Pseudobatos leucorhynchus

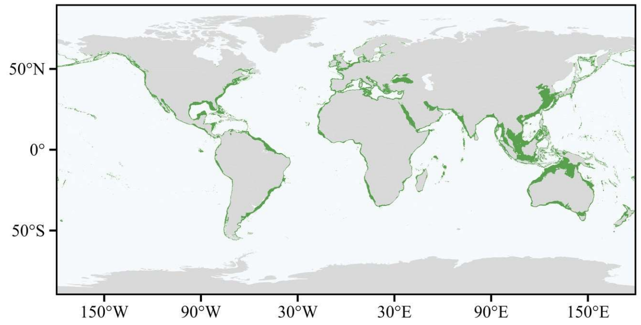

Rhinobatos albomaculatus

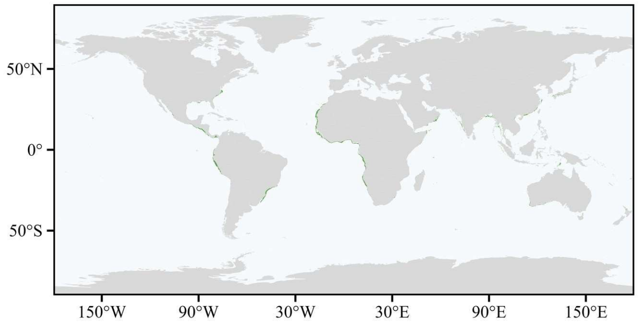

Rhinobatos rhinobatos

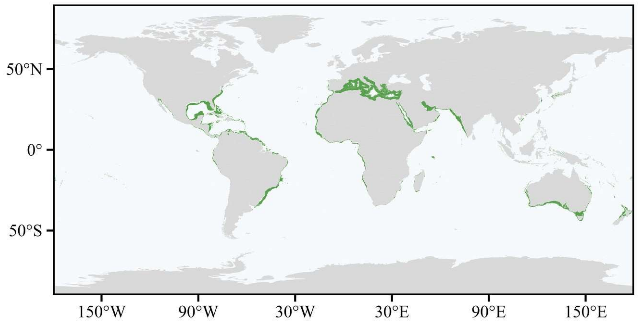

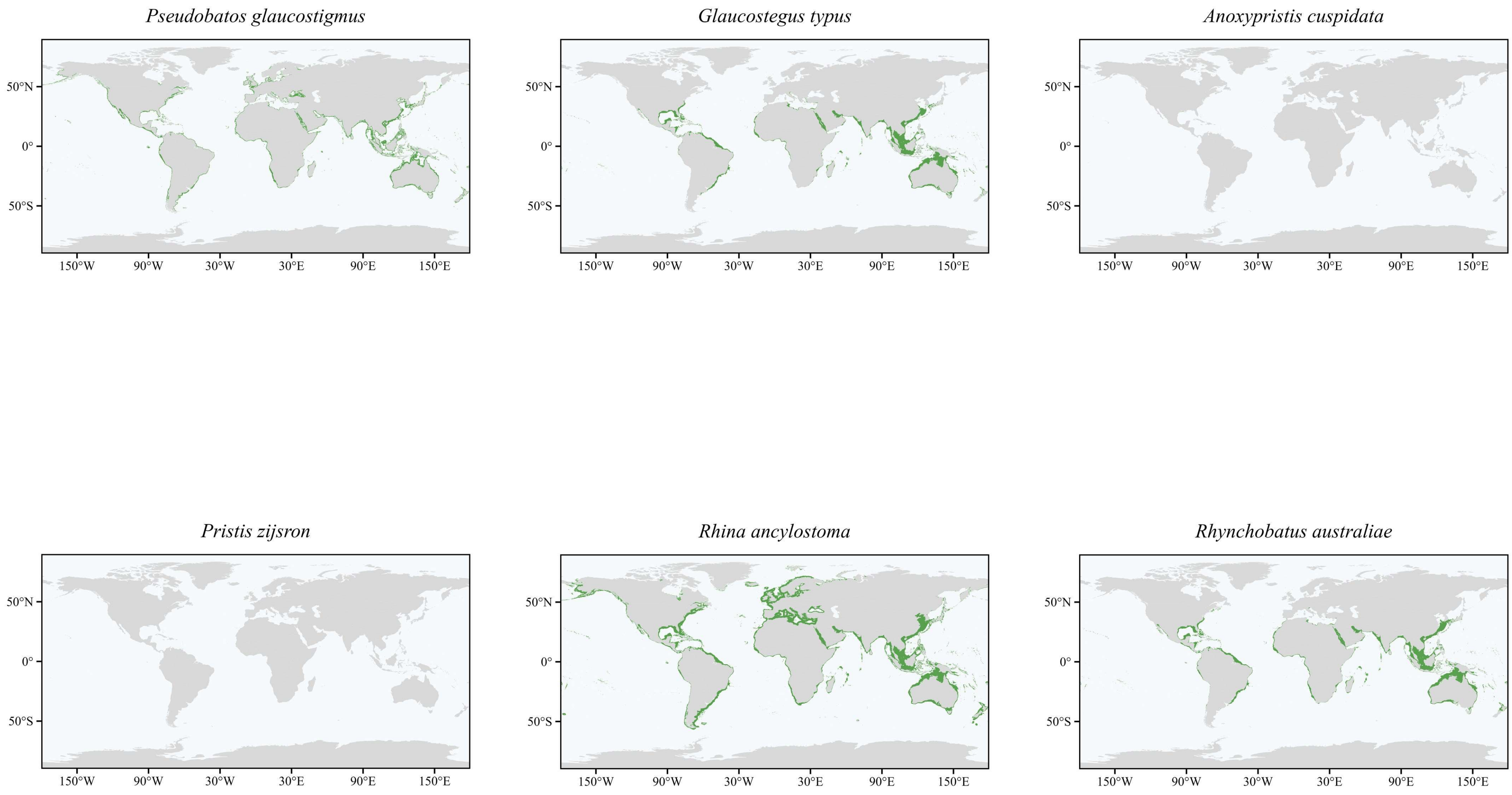
Pseudobatos glaucostigmus
Glaucostegus typus
Anoxypristis cuspidata
Pristis zijsron
Rhina ancylostoma
Rhynchobatus australiae
50°N
0°
50°S
150°W
90°W
30°W
30°E
90°E
150°E

Figure S2(b). Future binary suitable-habitat projections for Rhinopristiformes.

Rhynchobatus djiddensis

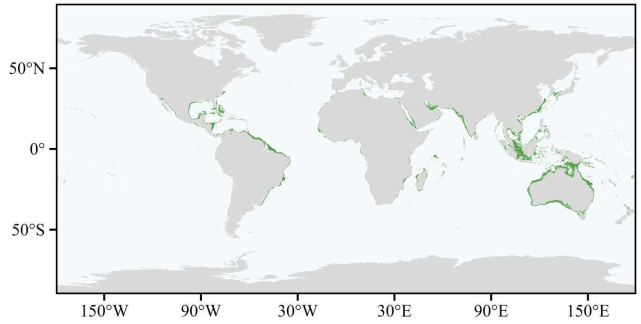

Zapteryx xyster

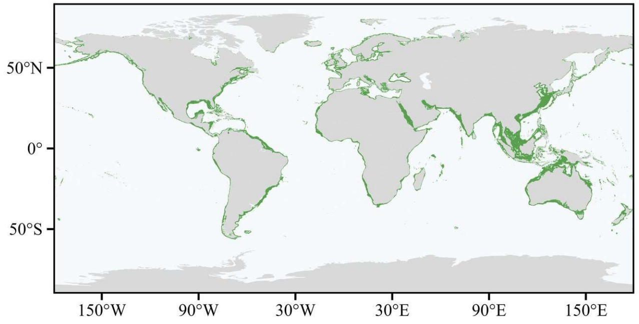

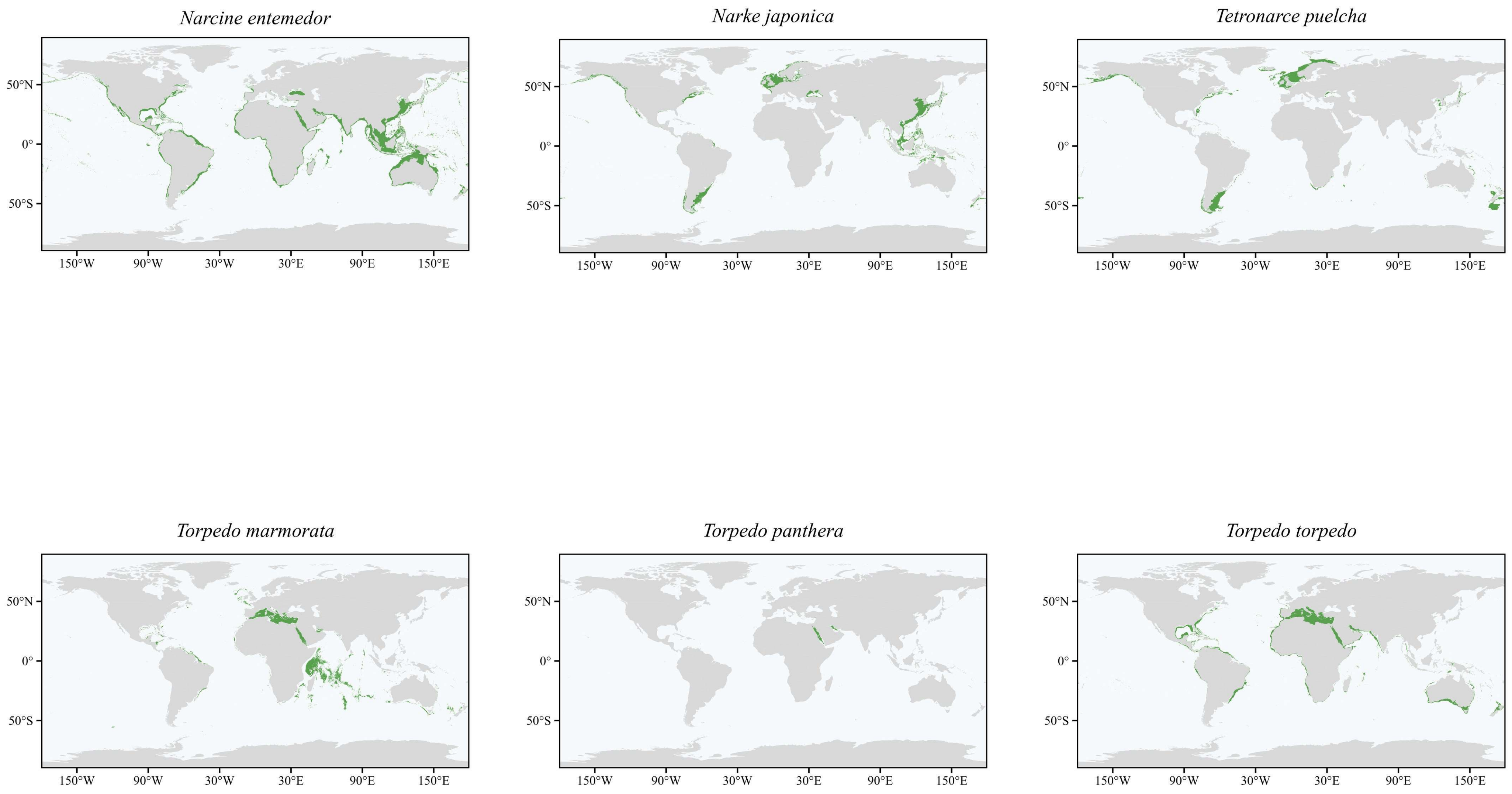
Narcine entemedor
Narke japonica
Tetronarce puelcha
Torpedo marmorata
Torpedo panthera
Torpedo torpedo
50°N
0°
50°S
150°W
90°W
30°W
30°E
90°E
150°E

Figure S2(d). Future binary suitable-habitat projections for benthic Myliobatiformes.

Bathytoshia centroura

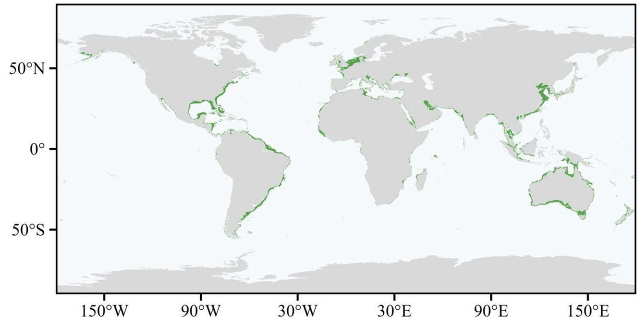

Bathytoshia lata

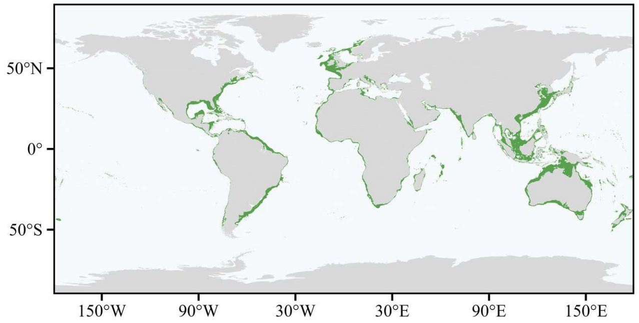

Dasyatis pastinaca

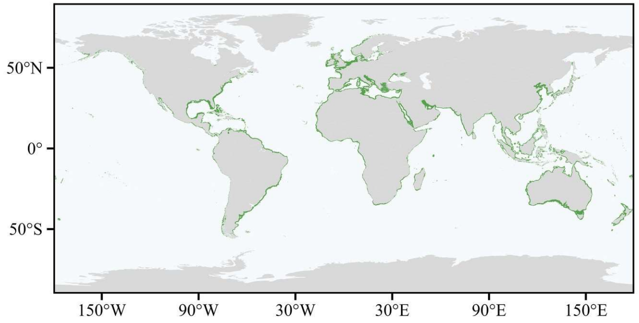

Himantura leoparda

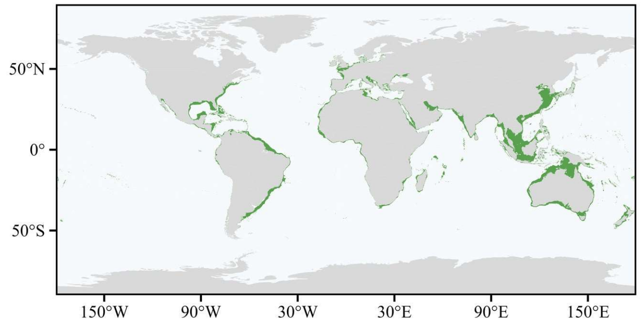

Himantura uarnak

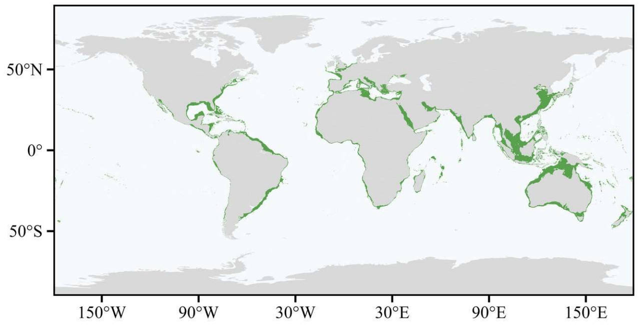

Hypanus berthalutzae

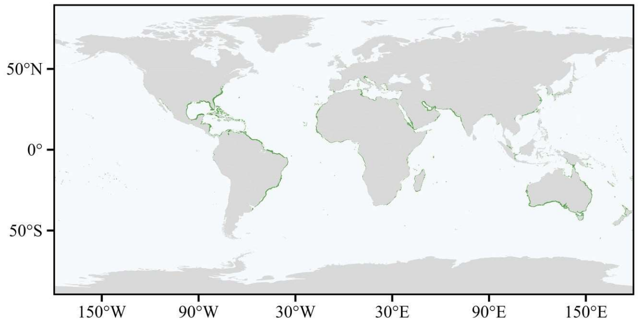

Hypanus dipterurus

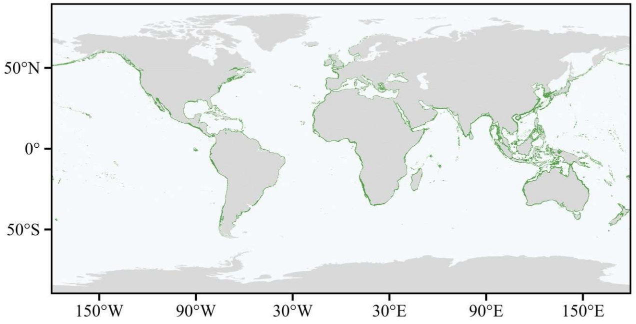

Hypanus longus

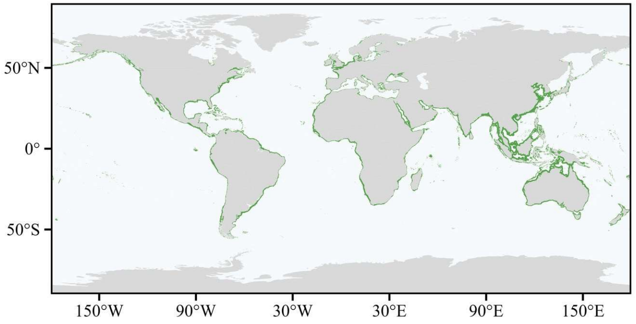

Maculabatis gerrardi

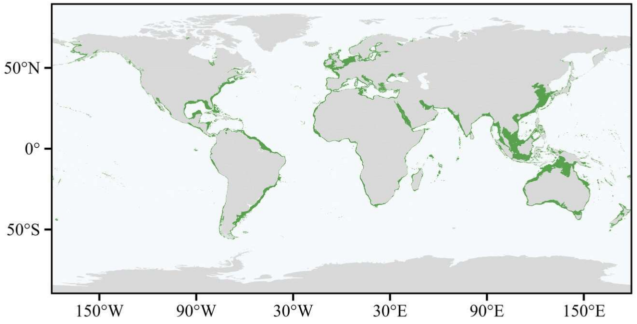

Pastinachus ater

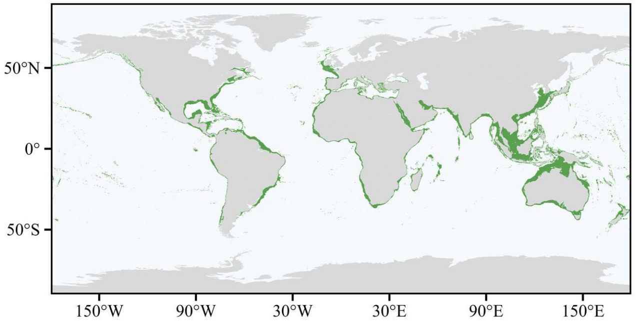

Pateobatis fai

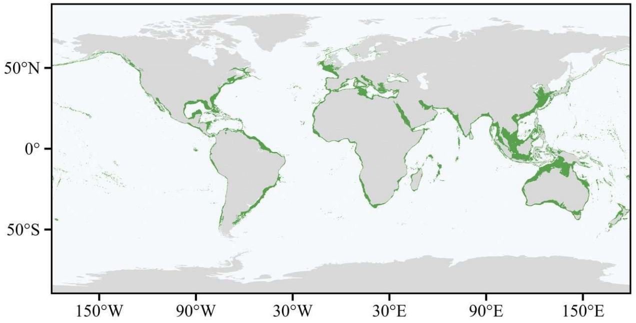

Pateobatis jenkinsii

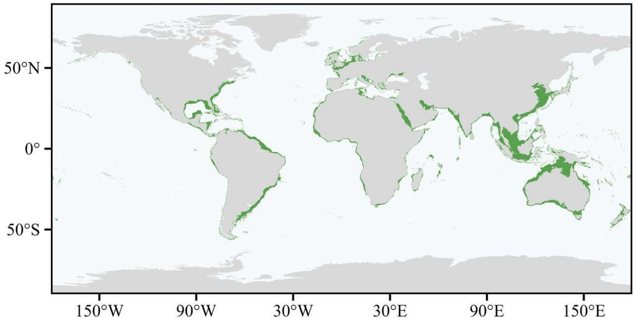

Taeniurops meyeni

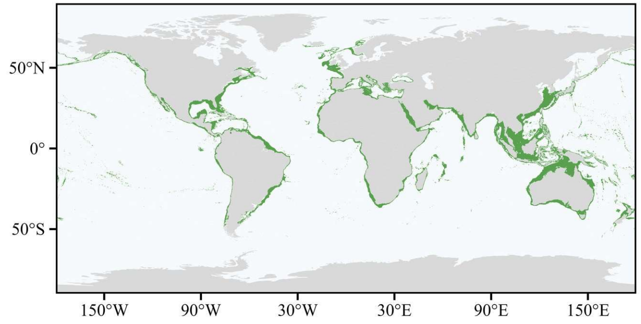

Telatrygon crozieri

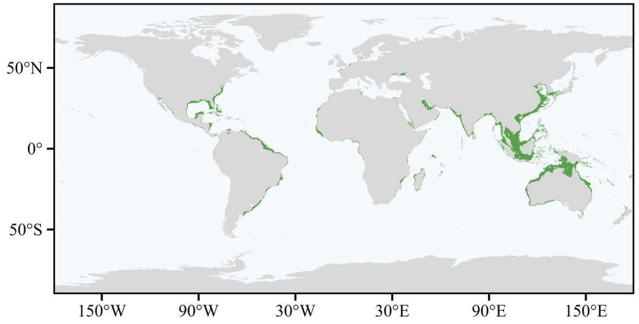

Telatrygon zugei

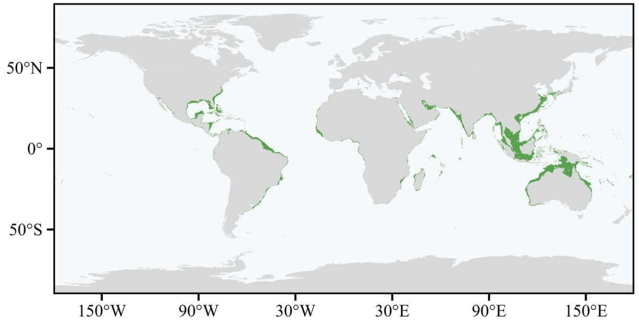

Urogymnus asperrimus

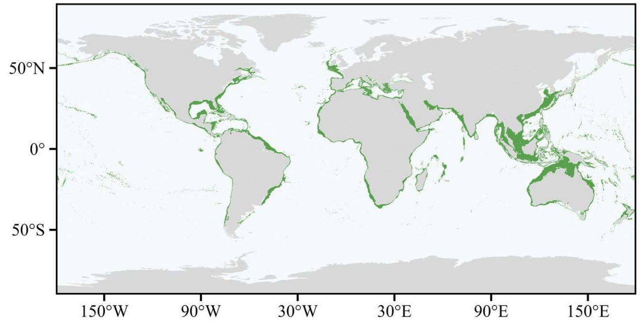

Urogymnus granulatus

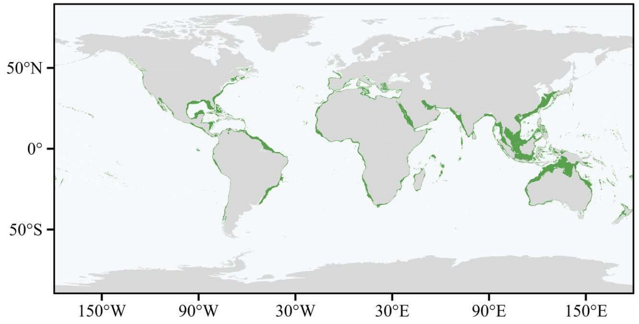

Urolophus aurantiacus

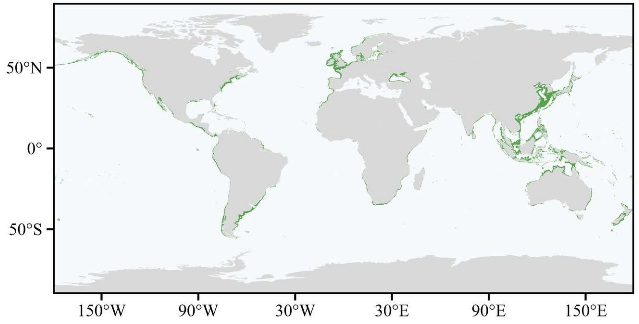

Figure S2(d). Future binary suitable-habitat projections for benthic Myliobatiformes.

Urolophus bucculentus

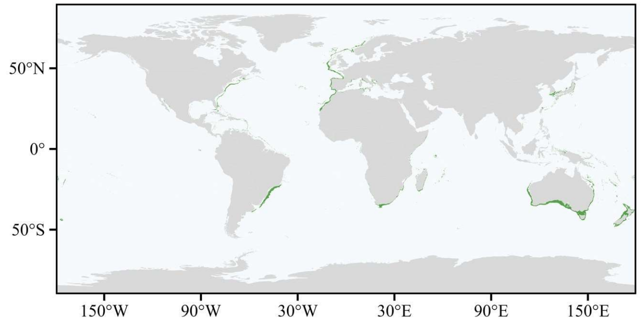

Urolophus sufflavus

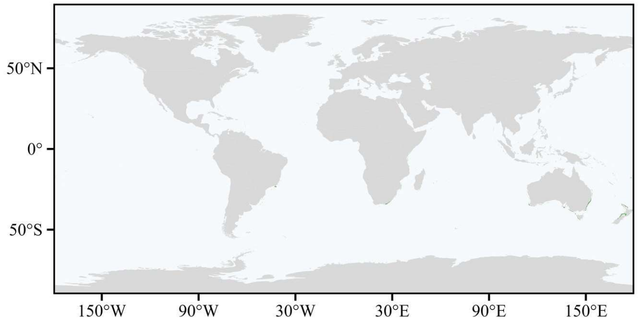

Urolophus viridis

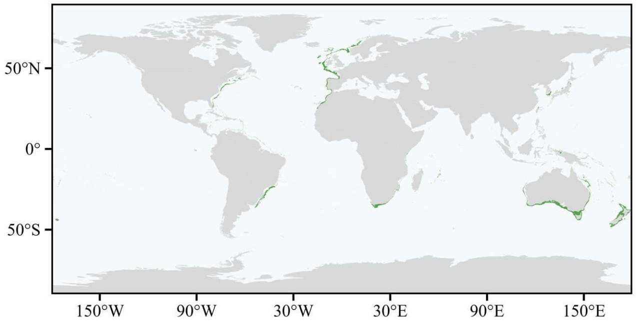

Gymnura altavela

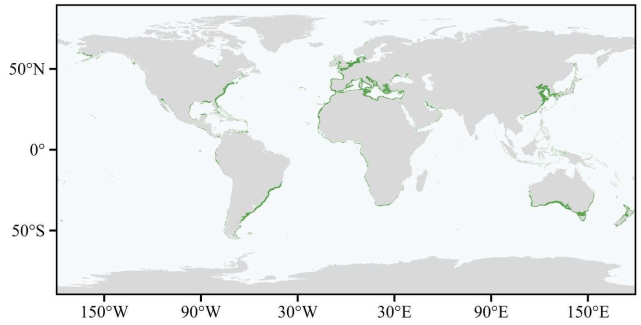

Gymnura poecilura

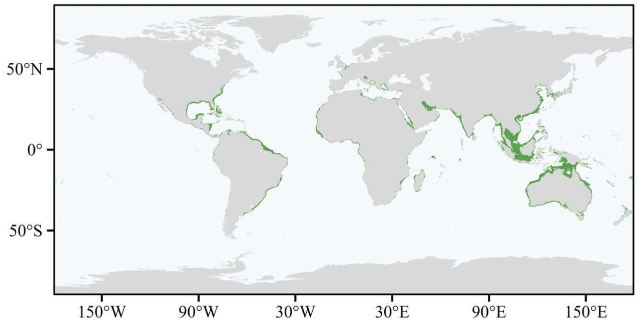

Figure S2(e). Future binary suitable-habitat projections for pelagic Myliobatiformes. *1-6 of 18 species*

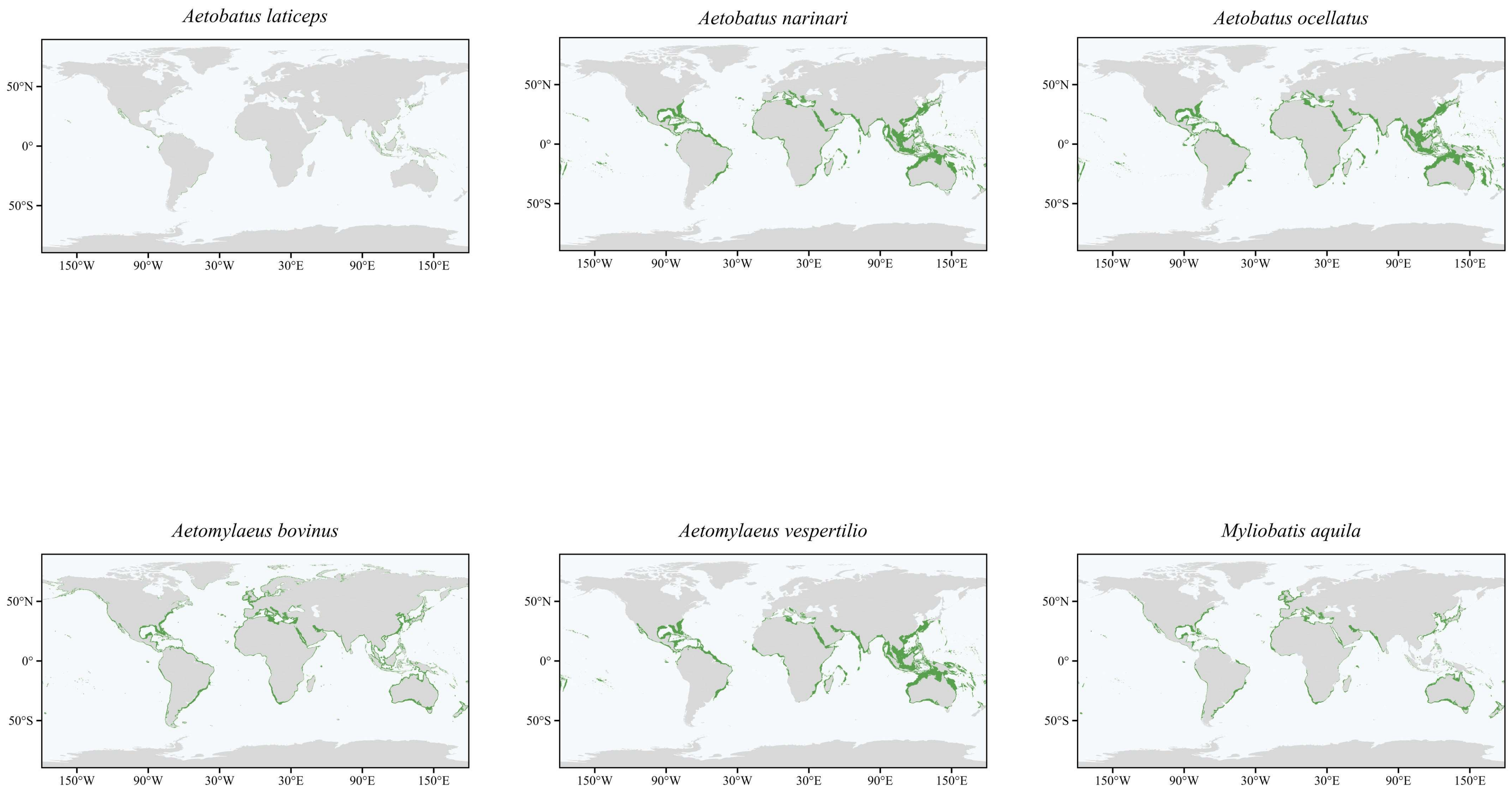

Figure S2(e). Future binary suitable-habitat projections for pelagic Myliobatiformes.

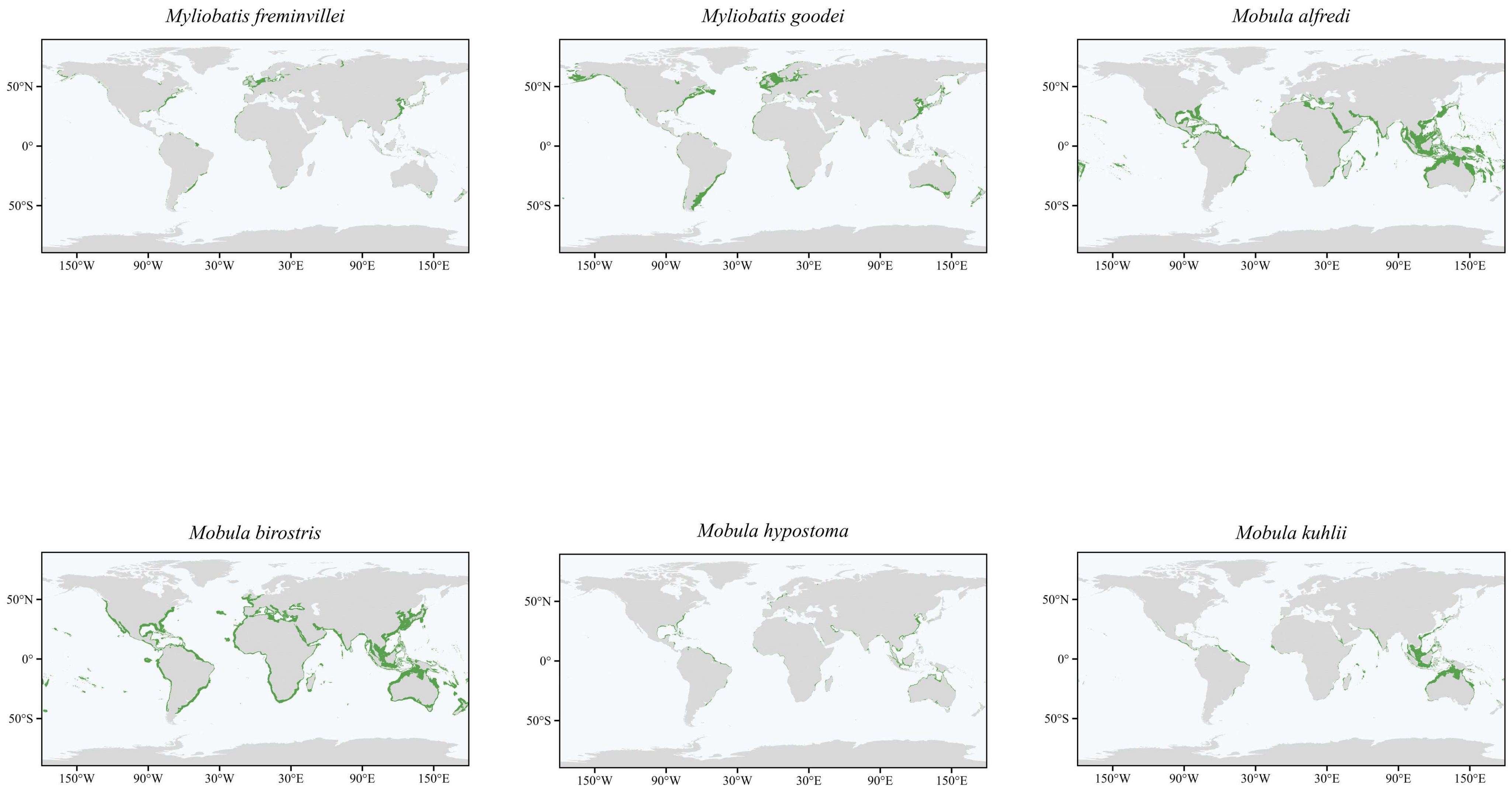

Mobula mobular

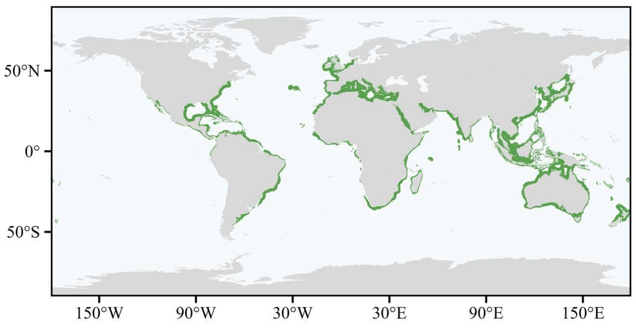

Mobula munkiana

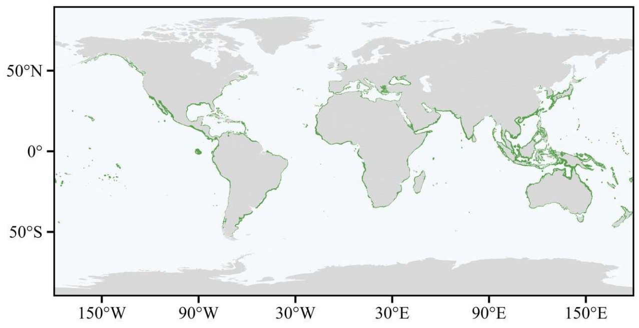

Mobula tarapacana

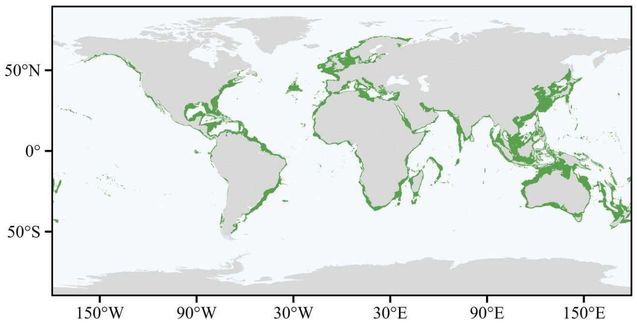

Mobula thurstoni

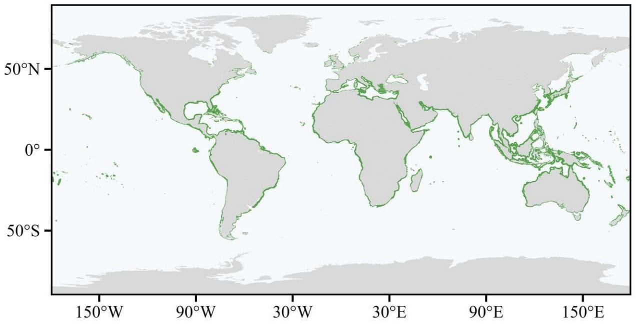

Rhinoptera bonasus

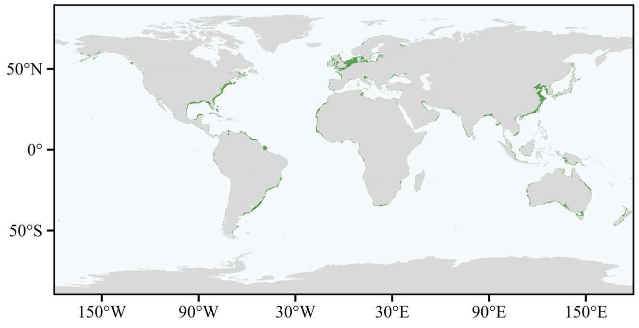

Rhinoptera javanica

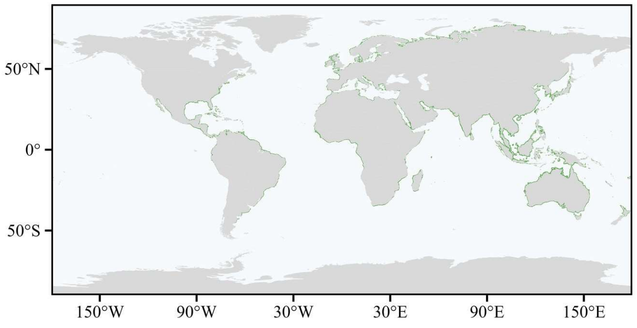

Supplement: Supplementary file 1 [file animals-16-02924-s001.zip › Figure_S2_species_level_future_binary_projections_SSP585_2090_2100.pdf]
